# Modeling Potential Habitat for *Amblyomma* Tick Species in California

**DOI:** 10.3390/insects10070201

**Published:** 2019-07-08

**Authors:** Emily L. Pascoe, Matteo Marcantonio, Cyril Caminade, Janet E. Foley

**Affiliations:** 1 Department of Medicine and Epidemiology, School of Veterinary Medicine, University of California, Davis, CA 95616, USA; 2 Department of Pathology, Microbiology, and Immunology, School of Veterinary Medicine, University of California, Davis, CA 95616, USA; 3 Department of Epidemiology and Population Health, Institute of Infection and Global Health, University of Liverpool, Liverpool CH64 7TE, UK; 4 Health Protection Research Unit in Emerging and Zoonotic Infections, University of Liverpool, Liverpool L69 3GL, UK

**Keywords:** *Amblyomma*, invasive species, MaxEnt, species distribution modeling, ticks

## Abstract

The *Amblyomma* genus of ticks comprises species that are aggressive human biters and vectors of pathogens. Numerous species in the genus are undergoing rapid range expansion. *Amblyomma* ticks have occasionally been introduced into California, but as yet, no established populations have been reported in the state. Because California has high ecological diversity and is a transport hub for potentially parasitized humans and animals, the risk of future *Amblyomma* establishment may be high. We used ecological niche modeling to predict areas in California suitable for four tick species that pose high risk to humans: *Amblyomma americanum*, *Amblyomma maculatum*, *Amblyomma cajennense* and *Amblyomma mixtum.* We collected presence data in the Americas for each species from the published literature and online databases. Twenty-three climatic and ecological variables were used in a MaxEnt algorithm to predict the distribution of each species. The minimum temperature of the coldest month was an important predictor for all four species due to high mortality of *Amblyomma* at low temperatures. Areas in California appear to be ecologically suitable for *A. americanum*, *A. maculatum,* and *A. cajennense*, but not *A. mixtum*. These findings could inform targeted surveillance prior to an invasion event, to allow mitigation actions to be quickly implemented.

## 1. Introduction

The *Amblyomma* genus of ticks comprises typically aggressive and generalist species of considerable human and veterinary medical importance [1]. In addition to injury and potential secondary infections associated with insertion of the long mouth parts of *Amblyomma* ticks [2,3], some species can transmit pathogens to humans and other animals [1]. In the USA, the lone star tick (*Amblyomma americanum*) often feeds on humans and can transmit *Ehrlichia chaffeensis* (causative agent of human monocytic ehrlichiosis), *Ehrlichia ewingii* (agent of human granulocytic ehrlichiosis), *Coxiella burnetii* (agent of Q fever), and *Francisella tularensis* (agent of tularemia) [4,5,6,7,8]. Though not apparently a vector of *Borrelia burgdorferi* sensu stricto (agent of Lyme disease) [9,10], *A. americanum* can transmit the southern tick-associated rash illness (STARI) pathogen, causing a disease clinically similar to Lyme borreliosis [11]. The Gulf Coast tick, *Amblyomma maculatum,* is an important vector of *Rickettsia parkeri* to humans [12,13] and *Hepatozoon americanum*, a pathogenic protozoan of dogs and wild canids [14]. The Gulf Coast tick can also transmit *Ehrlichia ruminantium* (agent of heartwater), which, although not currently present in the USA, could lead to high rates of livestock mortality if introduced [15,16]. Ticks in the *Amblyomma cajennense* species complex, including *A. cajennense* sensu stricto (further referenced as *A. cajennense*) and *Amblyomma mixtum*, are considered the most important vectors of *Rickettsia rickettsii* (agent of Rocky Mountain spotted fever) in Central and South America [17].

**Figure 1:**
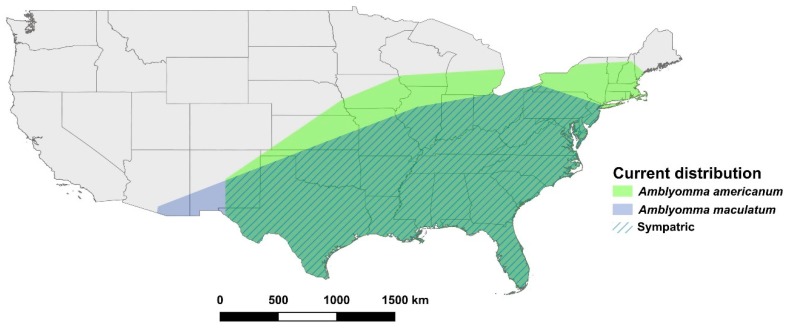
The current USA distributions of *Amblyomma americanum* (the lone star tick) and *A. maculatum* (the Gulf Coast tick) based on the generation of a convex polygon around geolocations for each species from the scientific literature and online databases collected for MaxEnt modeling (see Supplementary Materials for full list of geolocations).

Geographic ranges of *Amblyomma* species are rapidly expanding due to changes in climate and land-use, and increased movements and ranges of human and animal hosts [18]. Both *A. americanum* and *A. maculatum* are native to southeastern coastal USA, but are considered invasive due to their rapidly expanding westward and northward distributions, particularly *A. americanum* which now has established populations extending inland into midwestern states [19,20] (Figure 1). *Amblyomma cajennense* is currently only present in the southernmost states of the USA, and its range extends south to northern Argentina and east to the Caribbean, making it one of the most widely distributed ticks in the New World [21]. The native range of *A. mixtum* extends from Mexico and Central America to western Ecuador [22] and includes the West Indies [23]; this tick is now invasive in Texas [24]. However, despite multiple introductions of *A. americanum* into California (usually on imported cattle), there are currently no known established populations of *Amblyomma* spp. ticks in the state [25,26]. Nevertheless, California may be at risk of invasion due to high ecological diversity that already supports many other ticks and suitable host species [27,28,29,30]. In addition, California is a hub for vast global movements of people, animals, and goods, imposing a high likelihood of introduction of non-endemic ticks. For example, in 2017 approximately 675,000 cattle were imported into the state [31]. While efforts are made to prevent concurrent importation of ticks, with such large-scale movements of livestock, as well as people and domestic pets which do not undergo quarantine, introduction of *Amblyomma* species into California poses a real threat.

Targeted surveillance may be one of the most effective ways of preempting tick invasion. Surveillance can be informed by species distribution modeling (SDM), a method that predicts the possible range of a species based on ecological variables that sustain species survival. Similar modeling methods have successfully preempted tick establishment in novel ranges, for example the establishment of *Ixodes scapularis* in Canada [32,33], and the retrospective range expansion of *I. scapularis* across the eastern USA [34]. The potential distribution of *A. americanum* in the USA has been predicted using SDM methods [35,36,37,38], but typically only for eastern states or based on presence data at a coarse county-level spatial resolution. To the best of our knowledge, distributions of *A. maculatum, A. cajennense,* and *A. mixtum* have not been predicted for the USA using SDM methods. Here, we use presence data at high spatial accuracy coupled with SDMs to predict the potential distribution of *A. americanum, A. maculatum, A. cajennense,* and *A. mixtum,* given present environmental conditions in California in the event that these ticks are successfully introduced in the near future.

## 2. Materials and Methods

### 2.1. Amblyomma spp. Presence Data

Data on tick presence were obtained from the published literature and online databases. A search of the published literature for location data on *A. americanum, A. maculatum, A. cajennense,* and *A. mixtum* was conducted in the Web of Science core collection: for each tick all articles with the species or common name in either the topic or the title were retrieved. All relevant publications documenting tick presence and location were selected from the search results, as were the references within. When provided, latitude and longitude (or equivalent) coordinates were collected. If coordinates were not provided but information pertaining to administrative boundaries in the USA were (e.g., name of state park), the polygon constrained to this boundary was selected from a vector layer (e.g., the United States Geological Survey Protected Areas Database of the United States) in QGIS version 3.4 [39]. Vegetation and land cover type within the polygon was assessed using the National Land Cover Database (NLCD) 2011 [40]. The polygon was then constrained to incorporate only those land cover types either described to have been sampled within the publication, or which are described in the literature as being able to support the tick (e.g., [41,42,43]). The random points tool in QGIS was then used to select a random point within this constrained administrative boundary polygon. In areas where administrative boundary and/or NLCD data were not available (e.g., outside of the USA), but a detailed description of the location was provided, satellite images available at ©2019 Google Maps (https://www.google.com/maps/) [44] were used in QGIS using the OpenLayers application, to define the area boundary and vegetation suitability. The area was subsequently converted into a polygon and a random coordinate was selected within. If location information was only provided at a coarse-scale (e.g., county, region of a state), or it was not possible to accurately discriminate tick location, data were excluded.

**Figure 2:**
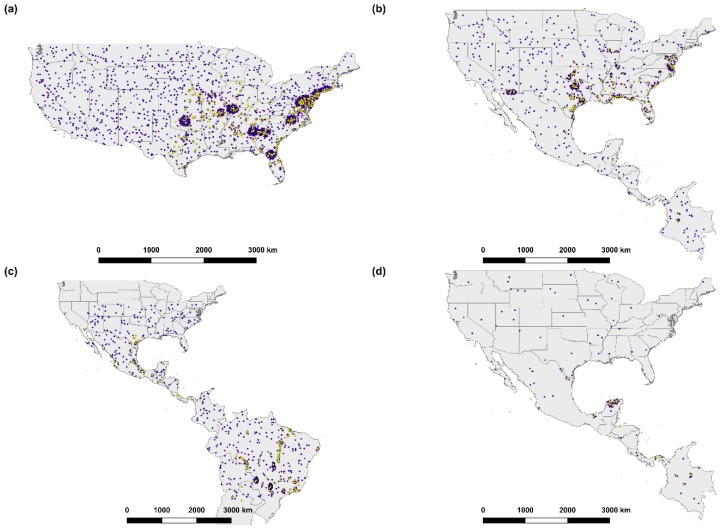
Geolocations used in MaxEnt modeling to predict suitable habitat in California for four *Amblyomma* tick species: (**a**) *A. americanum,* (**b**) *A. maculatum*, (**c**) *A. cajennense,* and (**d**) *A. mixtum*. Presence geolocations (yellow circles) were obtained from the published literature and open access databases, and background geolocations (blue squares) were simulated according to assumed presence sampling bias.

Location data were also obtained from the Global Biodiversity Information Facility (GBIF; https://www.gbif.org/), Biodiversity Information Serving Our Nation (BISON; https://bison.usgs.gov/#home), and VectorMap (http://vectormap.si.edu/ [Accessed January 14 2019], see [45,46,47] for original data sources pertaining to this database). Data submitted by BISON, research institutes, universities, or other scientific organizations were included, as were data from online identification databases, provided that the species identification had been verified by an expert (BugGuide and iNaturalist research grade observations). To be included in any model, all geolocation data had to satisfy the following quality control measures: be an observation from no earlier than 1950, include two decimal places or more for at least one coordinate, and have a coordinate inaccuracy of ≤1000 m. In addition, geolocations were cross-checked to ensure that they represented the site of observation described; for example, geolocations were omitted if they fell within a museum where a preserved specimen was kept (Figure 2, see Supplementary Materials for full list of geolocations).

Background geolocations (a proxy for species absence) were generated for each species at a ratio of 4:1 presence geolocations [48]. For each of the four species, 50% of background geolocations were generated randomly within the geographical extent of tick presence using the randomPoints function in the dismo R package [49]. For the other 50% of background geolocations, heatmaps based on kernel density estimates (KDE) with a 100 km bandwidth around presence geolocations were created in QGIS, and were subsequently converted into a polygon using the digital number (DN) value of pixels in the density kernel raster as polygon attributes. The random points tool was then used to randomly select coordinates within each polygon “biased” towards sampling efforts based on the standardized kernel density value (Figure 2). The use of random background geolocations assumes that all locations are equally likely to be invaded, as may be possible for *Amblyomma* ticks which could be introduced by long distance means, such as livestock importation or host migration. However, at the same time, using background geolocations based on KDE values acts to reduce oversampling bias in presence geolocations related to, for example, ease of accessibility to sampling sites [48]. To minimize pseudo-replication and oversampling bias, only one presence or background geolocation was permitted in each predictor raster pixel (~1 km^2^). This was achieved by excluding duplicated geolocations and using the gridSample function in dismo which randomly retains a single geolocation from each raster pixel [49].

### 2.2. Environmental Predictor Variables

Predictor variables included 19 bioclimatic variables, elevation, slope, and average and standard deviation of normalized difference vegetation indices (NDVI) (see Table 1 for full list of variables). Bioclimatic variables can indirectly affect habitat suitability for ticks, elevation impacts microclimate, host presence, and vegetation [50], whilst slope provides a proxy for the velocity of subsurface water flow and runoff rate, and thus can represent soil moisture content [51,52]. Suitability of tick habitat is also affected by the presence and type of vegetation (e.g., [53,54]), and vegetation greenness/biomass can be described using NDVI. Two different datasets were used to derive bioclimatic “Bioclim” variables: To train and test the models, we used WorldClim 2.0 at 30 arc seconds resolution [55], whereas the 4 km resolution parameter-elevation regressions on independent slopes model (PRISM) [56] dataset was used to predict potential habitat suitability in California. Both WorldClim and PRISM are a set of climate layers, such as precipitation and temperature, that are interpolated from weather station data [55], which may impact tick survival (e.g., [57]). The use of two datasets was necessary as WorldClim data only span the 1950–2000 period. Although WorldClim data geographically and temporally matched species observations, they would not provide recent climatic information. For prediction, monthly average minimum and maximum temperature and cumulative precipitation were downloaded from the PRISM website for 2014–2018. Long term monthly averages were then derived for each variable and used to calculate 2014–2018 Bioclim variables with the r.bioclim module in GRASS GIS 7.6 [58].

Elevation data were downloaded from the processed Consultative Group for International Agricultural Research-Shuttle Radar Topographic Mission dataset (i.e., data gaps filled by interpolation) available at 90 m resolution. Slope was computed from elevation data using the r.slope.aspect module in GRASS GIS version 7.4 [58,59]. No noise (smoothed) NDVI 4 km, 7-day composite data were downloaded from the National Oceanic and Atmospheric Administration STAR dataset for the years 1985–2000 (training and testing) and for 2014–2018 (prediction) (https://www.star.nesdis.noaa.gov/smcd/emb/vci/VH/VH-Syst_10ap30.php). NDVI is the difference between near-infrared light emissions which are reflected by vegetation and visible red light which are absorbed by vegetation. These data were imported into GRASS GIS version 7.64, and the mean and standard deviation were calculated. All training and prediction environmental variables were re-sampled using bilinear spatial interpolation to standardize resolution to 30 arc seconds, imported into R using the raster package (version 2.6-7 [60]), and cropped to the extent and shape of the countries from which *Amblyomma* tick species presence data were obtained.

Predictor variable values were extracted for each presence and background geolocation (30 arc seconds pixel). Many presence and background geolocations near the coast were not well covered by the extent of the environmental predictor raster files; therefore, when a raster did not overlap a geolocation and environmental data could not be extracted a distance matrix was applied to sample the nearest non-empty pixel within a 10 km radius. For data extracted for presence geolocations, collinearity between each pair of predictor variables was assessed using Pearson’s correlation coefficients. When the absolute value of the correlation coefficient was ≥0.80, we retained just one of the two variables in the model. This selection was informed by: the variable with the highest percent contribution to the model (that is, in each iteration of the training algorithm, the increase in regularized gain was added to the contribution of the corresponding variable, or subtracted from it if the change to the absolute value of lambda was negative), and the ecological relevance to the tick species of the variable. Environmental predictor variables were refined until the model consisted of only non-correlated, biologically relevant variables and MaxEnt auto-feature classes were used for all species.

### 2.3. Modeling of Habitat Suitability

Models for each of the four *Amblyomma* species were run using the MaxEnt algorithm in the dismo package in R [49,61]. The MaxEnt algorithm uses machine learning to make predictions based on data that lacks true absence records. The algorithm is deterministic and uses maximum entropy to estimate the most uniform distribution of presence geolocations compared to background geolocations, within the constraints derived from the environmental predictor data [61]. The output of model convergence describes how much better the model fits the presence data compared to a uniform distribution of the species throughout the study extent [61,62]. Model inputs were the environmental predictor variable data extracted from *Amblyomma* spp. presence and background geolocations. Following optimization, model regularization (betamultiplier) was set at 5 to minimize model over-fitting.

### 2.4. Model Evaluation and Visualization

To test the performance and reduce variance of the final MaxEnt model, we used a 10-fold cross-validation method: A seed was set (to allow repeatability) before both presence and background data were each randomly partitioned into 10 equal subsets comprising one test subset and nine training subsets. The model was iterated 10 times using a different test and training subset each time [63,64]. The i) MaxEnt output percent contribution and ii) permutation importance of each environmental predictor variable in the models, as well as iii) habitat suitability predictions, were averaged (mean) over the 10 model iterations to reduce variance [63,65]. Model performance was evaluated using the average area under the curve (AUC) of receiver operator characteristics from testing of the ten models. AUC values from 0–0.5 are considered to have predictive probabilities no better than random, and values from 0.5–1 indicate that the model has greater power to predict high habitat suitability in locations of known presence, whereby the closer to 1 the value is, the more “perfect” the prediction [61].

Response plots indicating the correlation between environmental variables with habitat suitability were derived using the ratio of probability density for each predictor at presence to background geolocations, considering data from all the 10 models used for model training and testing. Curves were then smoothed using LOESS (locally estimated scatterplot smoothing) with span = 1. We converted each continuous surface for the averaged-model prediction into a binary representation of suitable habitat using a probability threshold that represented maximum specificity at maximum sensitivity (1.00). That is, at the selected threshold, all tick training presence geolocations were correctly classified (no omission) as suitable by the model, at which the maximum background geolocations were correctly classified. The resulting binary raster classified each 1 km^2^ pixel as either suitable or unsuitable, with a score of 1 or 0, respectively. This raster was used to map the predicted habitat suitability for *Amblyomma* ticks in California. 

## 3. Results

### 3.1. Amblyomma americanum

**Figure 3:**
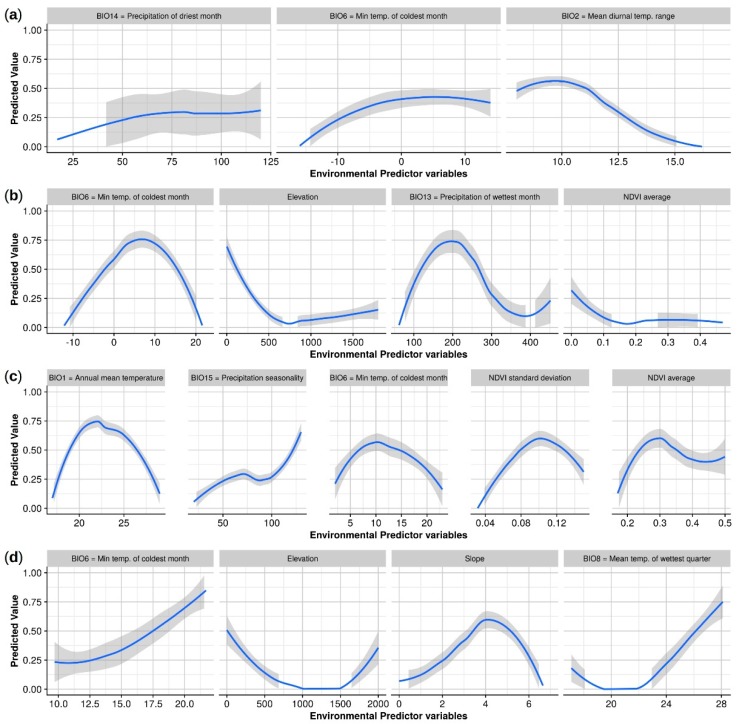
Estimated smoothed trends (using locally estimated scatterplot smoothing (LOESS) with span = 1) and 95% confidence intervals between environmental predictor variables and predicted habitat suitability in MaxEnt models for four exotic *Amblyomma* species: (**a**) *A. americanum*; (**b**) *A. maculatum*; (**c**) *A. cajennense*; and (**d**) *A. mixtum.* Trends were derived from the ratio of probability density of each predictor at presence to background geolocations, considering data from 10 iterations used for model training and testing.

**Figure 4:**
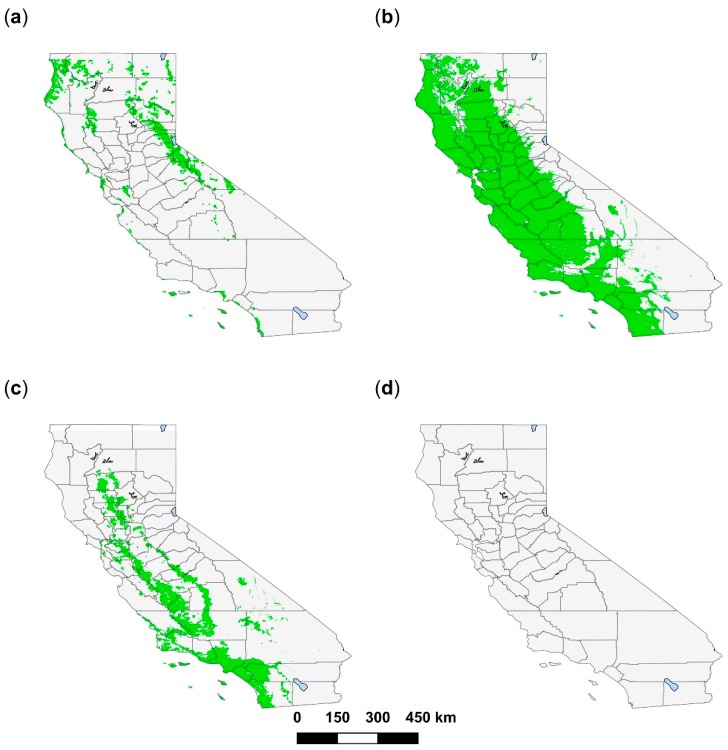
Habitat in California predicted (using MaxEnt species distribution modeling) to be environmentally suitable for four exotic *Amblyomma* tick species: (**a**) *A. americanum*; (**b**) *A. maculatum*; (**c**) *A. cajennense*; and (**d**) *A. mixtum*. Areas shaded in green represent potentially suitable habitat at a probability threshold at which all tick training presence geolocations were correctly classified as suitable by the model and which maximized correct classification of background geolocations. Hot arid desert was masked from suitability for *A. maculatum* and *A. cajennense*.

**Figure A1:**
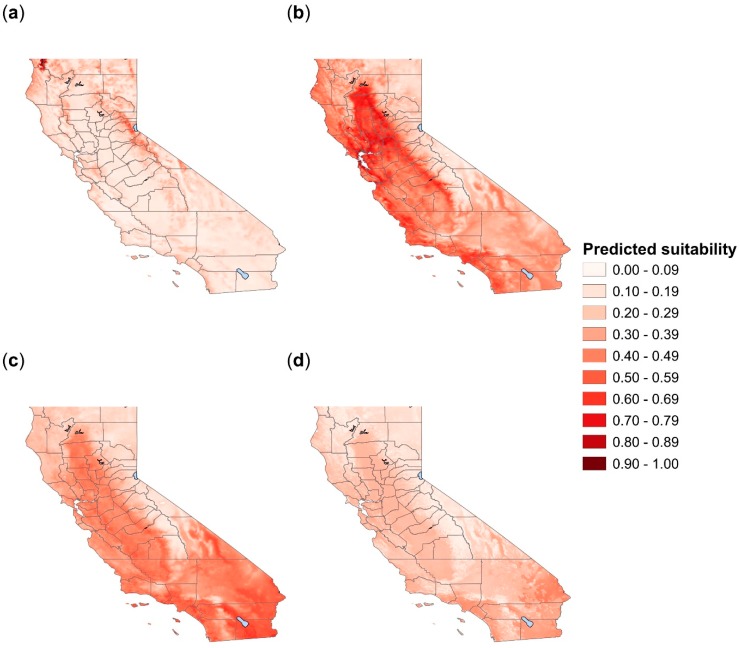
Heatmap of habitat suitability in California for four exotic *Amblyomma* tick species, predicted using MaxEnt species distribution modeling: (**a**) *A. americanum*; (**b**) *A. maculatum*; (**c**) *A. cajennense*; and (**d**) *A. mixtum*.

From an initial 912 *A. americanum* geolocations, 492 1 km^2^ presence pixels were retained in the model. The optimal model had a mean AUC of 0.78 (following 10 iterations) and included precipitation of the driest month (which contributed 59.9% to the model), minimum temperature of the coldest month (27.3%), and the mean diurnal temperature range (12.8%). Habitat suitability was positively correlated with precipitation of the driest month, with suitability peaking at approximately 75–100 mm precipitation (Figure 3). Suitability was also positively correlated with minimum temperature of the coldest month; where the minimum temperature was −15 °C habitat was not suitable, but suitability increased above this temperature to peak at 0 °C. The mean diurnal temperature range was negatively correlated with suitability, such that suitability was less than 50% when day-time temperature ranges exceeded 11 °C. Our model predicted suitable habitat for *A. americanum* along the length of the Californian coast and the San Francisco Bay area (Figure 4), due to mild minimum temperatures during the coldest month that rarely fall below 5 °C, and optimal diurnal temperature ranges (0–7 °C). Precipitation during the driest month is low throughout most of California, but throughout the Sierra Nevada mountain range, in some coastal counties in the north of the state (particularly Humboldt, Del Norte and Siskiyou (see Appendix A: Figure A1)), and the north Central Valley Tehama and Glenn Counties, there is at least 2 to 15 mm rainfall even during the driest months. These conditions are enough to provide patchy suitable habitat for *A. americanum*.

### 3.2. Amblyomma maculatum

From an initial 326 *A. maculatum* geolocations, 182 were retained in the model. The model had an AUC of 0.74 and incorporated four environmental predictor variables: minimum temperature of the coldest month (38.4%), elevation (30.6%), precipitation during the wettest month (18.9%), and average NDVI (12.2%) (Table 1). Suitability rose from 0%–75% between −10 and 7 °C in the coldest month before gradually declining at temperatures >7 °C (Figure 3). Suitability increased with precipitation of the wettest month between 0–200 mm but declined at >200 mm precipitation. Both elevation and average NDVI were negatively correlated with habitat suitability. Large swathes of California were predicted to be suitable for this species, including all of the Central Valley and the majority of the coast (Figure 4, see also Appendix A: Figure A1). These suitable areas have >70 mm of precipitation during the wettest month and mild temperatures during the coldest month which are rarely <0 °C. Most predicted suitable habitat was at elevations <500 m elevation, although some habitat did occur at up to 1500 m. The average NDVI was low in areas of predicted habitat (<0.4). Suitable habitat was predicted in the Death Valley (Appendix A: Figure A1); however, as this region is unlikely to be suitable for *A. maculatum,* due to an absence of vegetation and temperatures that exceed those which the tick can survive, this was removed from the binary map (Figure 4) by selecting and removing the arid, desert, hot as categorized by the Köppen-Geiger climate classification for California [66].

### 3.3. Amblyomma cajennense

From an initial 251 *A. cajennense* geolocations, 196 were retained in the model. The model had an AUC of 0.64 and was based on six environmental predictor variables (Table 1). Annual mean temperature contributed most to the model (68.2%), followed by precipitation seasonality (12.4%), minimum temperature of the coldest month (8.8%), and both the NDVI standard deviation (7.4%) and average (3.3%). Peak suitability occurred where annual mean temperature was approximately 23 °C and minimum temperature of the coldest month was 10 °C (Figure 3). Precipitation seasonality (that is, the temporal distribution of precipitation throughout the year) was positively associated with *A. cajennense* habitat. Suitability peaked at approximately 0.10 NDVI standard deviation (proxy for increased land-use change) and 0.30 average NDVI. A large portion of the southern Californian coastline, extending inland some 100 km, was predicted to be suitable for *A. cajennense*, as were the outer margins of the Central Valley (Figure 4, see also Appendix A: Figure A1). This coincides with areas that have a combination of relatively high mean annual temperatures (15–20 °C) and mild temperatures during the coldest month (>3 °C). Although the amount of precipitation varies temporally (seasonality) throughout the state, areas predicted to be suitable for *A. cajennense* had the highest variation in precipitation within California. NDVI standard deviation tended to be low in most of the state (≤0.2), but suitable areas were predicted in locations where the standard deviation was at the high end of its range and where overall NDVI average values were low. Suitable habitat was predicted in the Death Valley (Appendix A: Figure A1); however, as this region is unlikely to be suitable for *A. cajennense,* due to insufficient vegetation and temperatures that exceed those which the tick can survive, this was removed from the binary map (Figure 4) by selecting the arid, desert, hot as categorized by the Köppen-Geiger climate classification for California [66].

### 3.4. Amblyomma mixtum

All 30 geolocations collected for *A. mixtum* were used in the MaxEnt model. The model had an AUC of 0.73 and included four environmental predictor variables: minimum temperature of the coldest month, which contributed 79.6% to the model; mean temperature of the wettest quarter (14.3%); slope (5.3%); and elevation (0.7%). There was a positive association between the minimum temperature of the coldest month and habitat suitability, whereby at 10 °C suitability was 25%, increasing to 75% at >20 °C (Figure 3). Suitability was optimal from 0 to 500 m of elevation, and declined at elevations higher than this, although did increase again at elevations greater than 1500 m. An intermediate slope was most suitable for *A. mixtum*. Generally, there was a positive correlation between mean temperature of the wettest quarter and suitability. At the maximum sensitivity and specificity threshold, no habitat in California was predicted to be suitable for *A. mixtum* (Figure 4), with suitability predicted to be highest, but no greater than 50%, in southeast San Bernardino County (see Appendix A: Figure A1).

## 4. Discussion

We used the MaxEnt algorithm to predict habitat in California that could potentially support *Amblyomma* spp. tick vectors of medical and veterinary importance and that are undergoing range expansion [19,21,22,53,67]. A large area was predicted to be suitable for *A. maculatum* in California, a moderate extent was predicted for *A. americanum* and *A. cajennense,* but there was no predicted suitable habitat for *A. mixtum*. All models performed better than random (AUC ≥ 0.64).

Suitable habitat for *A. americanum* along the coast of California and the San Francisco Bay area was associated with mild minimum temperatures during the winter, moderate absolute annual temperatures, and adequate humidity. Mortality for this species is high at temperatures ≤–3.5°C [68], but between 2014 and 2018, average temperatures along most of the California coast did not fall below 5 °C. *Amblyomma americanum* is unable to withstand high temperatures, with a critical transition temperature (the threshold at which the cuticular lipids of the tick “melt” and are no longer able to prevent water loss, leading to desiccation) of 30–35 °C [69], constraining habitat to the mild and narrow temperature range such as occurs along the Californian coast. This species also requires high humidity [69,70], which occurred on the coast but also resulted from high levels of summer precipitation in the north of California and in the Sierra Nevada. Human pathogens for which *A. americanum* is a competent vector have been reported from areas of coastal and northern California predicted to be suitable for the tick, including *E. chaffeensis* [71] and *F. tularensis* [72].

Predicted habitat was widespread for *A. maculatum* in California, mostly in areas with high levels of precipitation during the wettest month and mild winter temperatures, occurring at elevations below 1500 m. Of the four species considered, this tick is likely to thrive best if introduced to the state. Because this tick is a vector of *R. parkeri,* its establishment in California could also result in the spread of this human pathogen, which until now has not been detected in the state. In contrast, DNA of *H. americanum* has been detected in dogs in California [73] and this canine pathogen could expand in range and prevalence if *A. maculatum* were established. It is necessary to consider that the accuracy of our predictions are contingent on the quality of the presence data we collected. *Amblyomma maculatum* is a member of a group of three morphologically similar species: *A. maculatum* sensu stricto (s.s.), *Amblyomma triste,* and *Amblyomma tigrinum,* and it is possible that some records we utilized were misidentified as *A. maculatum* s.s. [74]. We minimized this issue by restricting geolocation data for *A. maculatum* to the USA, Mexico, Belize, and Colombia, all of which are within the distribution of the *A. maculatum* s.s. species [75,76]. *Amblyomma tigrinum* does not typically occur within these four countries, although *A. maculatum* s.s. and *A. triste* do have sympatric distributions in some parts of northern South America including Colombia [74], whilst the population of *A. maculatum* described in Arizona is allopatric and inhabits very different habitat, which has led to speculation that this may be yet another species. Therefore, *A. maculatum* predictions should be interpreted with caution. However, because presence data used in the model corresponded to ticks able to vector pathogens of human and veterinary importance, albeit possibly not well-resolved to species, our model predictions are expected to be valid for risk of such medical significance in California.

The areas predicted to be suitable for *A. cajennense* have relatively high average annual and winter temperatures and are amongst some of the most densely populated areas of California. Typically, *A. cajennense* inhabits the equatorial Amazonian biome or in the USA where mean temperature ranges from between 13 and 16 °C, NDVI is high, and the climate is humid [21,22,23,77]. This tick has also been reported to favor disturbed or fragmented habitats [78]. *Amblyomma cajennense* is one of the most important vectors of *R. rickettsii* [17] in South America. This potentially fatal human and canine pathogen is emerging in northern Mexico, where it is associated with the brown dog tick, *Rhipicephalus sanguineus* [79,80]. Thus, the potential establishment of *A*. *cajennense* in California is a major public and animal health concern. Habitat suitability was highest in areas of California with high NDVI standard deviations (land-use change) and low NDVI average values compared to other parts of the state. These areas are largely rural and are unlikely to have undergone notable anthropogenic changes (such as deforestation, urbanization, reforestation, etc.); however, between 2011 and 2017 California experienced widespread drought [81,82], and as habitat suitability predictions were made based on 2014–2018 NDVI values, these would have captured the recovery of vegetation in 2017–2018 after the drought ended. Like *A. maculatum*, *A. cajennense* was previously considered a single species, but is now acknowledged as a species complex comprising at least six sub-species including *A. cajennense* sensu stricto (s.s.) and *A. mixtum* [21,22]. As with *A. maculatum,* every effort was made to include only those records referring to the *A. cajennense* s.s. species; however, it is possible that some reported presence data were instead representative of another species within the complex, and predictions for this species should be interpreted with caution.

The lack of suitable habitat for *A. mixtum* in California appeared to be mostly due to low minimum temperatures during the coldest month and high elevations in the state. This species is distributed between Texas and western Ecuador and appears to favor humid, tropical climates, but modeling for South and Central America has documented that habitat can be arid or moist if it has high, sustained temperatures throughout the season [77]. Additionally, this tick is considered one of the most desiccation-tolerant of the ixodids [83]. However, despite a mean AUC indicating that the model had good predictive power, this value varied greatly between the 10 model iterations. In addition, the response curve illustrating the correlation between elevation and *A. mixtum* habitat suitability showed an unusual relationship: Suitability decreased at >500 m elevation, but began to increase again at >1500 m. The variability in model performance and unusual response curves are likely due to the fact that our model was based on only 30 presence geolocations, which are unlikely to have represented the full range of environmental conditions suitable for this tick.

The current study predicted suitable *Amblyomma* habitat using environmental variables which included 19 climatic variables, elevation and slope, and mean and average of NDVI. The set of climatic variables were interpolated from weather station data, and included precipitation and temperature [55], which may impact tick survival (e.g., [57]). Elevation impacts microclimate, host presence, and vegetation [50], whilst slope provides a proxy for soil moisture content because it influences velocity of surface and subsurface water flow [51,52]. A more direct assessment of vegetation, which influences ticks and wild animal hosts (e.g., [53,54]), is provided by NDVI. The average NDVI provides information on the greenness of vegetation, while the standard deviation can be used as a proxy for the magnitude of land-use change [84,85]. Environmental predictor variables were standardized to a resolution of 30 arc seconds, which is equivalent to approximately 1 km^2^ at the latitude of California. However, there is some mis-match in spatial scales, as ticks are also affected by microclimatic variables that vary on a much finer spatial scale ranging from a few centimeters to meters, for example soil temperature and humidity, which may vary markedly within an area [86,87].

Our models did not explicitly include availability of preferred domestic and wild hosts of the ticks, although the four *Amblyomma* species in our study tend to feed on diverse host species. *Amblyomma americanum* adults often feed on white-tailed deer (*Odocoileus virginianus*) and the closely related mule deer (*Odocoileus hemionus*) is abundant in California, as are other medium and large mammals that this tick feeds on, such as raccoon (*Procyon lotor*) [18]. In the Midwest USA, *A. americanum* is referred to as the “turkey tick” due to its propensity to parasitize wild turkeys (*Meleagris gallopavo*) [18], a bird that is widespread in California. *Amblyomma maculatum* has been recorded as parasitizing over 60 different host species, including common rural and urban species in California such as the fox squirrel (*Sciurus niger*), coyote (*Canis latrans*), and house wren (*Troglodytes aedon*) [19]. Both *A. cajennense* and *A. mixtum* typically feed on large mammals, particularly ungulates [21]. Feral swine (*Sus scrofa* spp.) are abundant in California, and host *A. americanum*, *A. maculatum*, and *A. cajennense* in other states including Texas [88,89], and could serve as vehicles of introduction, as could parasitized migratory birds that spend time in California, such as the white-throated sparrow (*Zonotrichia albicollis*) and Lincoln’s sparrow (*Melospiza lincolnii*) [90]. The model did not directly include organic matter in the soil, and litter and duff layer density [87,91] as environmental predictor variables. Although NDVI provides an estimate of vegetation greenness/biomass, the parameters did not distinguish between species of vegetation, some of which may impact habitat suitability for the tick. For example, soil surface is hotter and drier in the presence of Japanese stiltgrass (*Microstegium vimineum*), which is invasive in the USA, causing significant reductions in *A. americanum* survival [92]. On the other hand, shrubby vegetation, in particular, has been linked with increased *A. americanum* and *A. maculatum* abundance, and forest is preferred to grassland [41,42,43]. Presence and establishment of these ticks may be impacted by vegetation succession, particularly in areas disturbed by fire or flooding (e.g., in the Coastal mountain range in north California, which has suffered both extensive fires and flooding in recent years).

Southern California, at the borders of Arizona and Mexico, appears particularly vulnerable to introduction of *Amblyomma* spp. ticks. *Amblyomma maculatum* habitat was predicted near the California–Arizona border, approximately 400 km from sites already colonized in Arizona [93] (see also BISON and GBIF records), and *A. cajennense* has been documented <1 km from the Mexico–California border (GBIF records). Invasion of *A. maculatum* and *A. cajennense* into California could be facilitated by the frequent local movements of humans, livestock, and wildlife in these areas. Even though predicted habitat in California for *A. americanum* and *A. cajennense* was patchy, movement of humans and livestock throughout the state could promote subsequent invasion events to suitable areas. In contrast, suitable habitat for *A. maculatum* was contiguous, possibly allowing a single invasion event to result in widespread colonization in California by these ticks.

*Amblyomma* ticks have been introduced to California in the past [25,26], and human and livestock movements have intensified in recent years, thereby increasing introduction risk. Although tick surveillance is performed throughout the state by researchers, public health agencies, and the public, these efforts are typically focused in areas where species posing a public health threat are already established. It does not necessarily reflect locations that may become infested with exotic ticks. The models presented here draw attention to the need to disseminate data on tick presence and on the introduction risk of exotic ticks. While tick establishment per se would not ensure introduction or spread of pathogens, the high vector competence of *Amblyomma* tick species for multiple pathogens could increase the burden of disease on public health in California. Successful tick introduction and establishment would require sufficient propagule pressure, introduction at a site with suitable habitat, and the presence of suitable host species. As ticks expand in distribution and are introduced to new areas, the chances of successful establishment are increased. Unified tick surveillance efforts would allow us to identify areas not being surveyed for ticks and to rapidly disseminate occurrences of all tick species, including those which are invasive.

## 5. Conclusions

We predicted that there is habitat that could support *A. americanum*, *A. maculatum,* and *A. cajennense* in California based on a set of environmental variables. These data can inform targeted surveillance prior to an invasion event, which is possible in California due to frequent and widespread movements of humans and animals (both wild and domesticated) into and around the state.

## Figures and Tables

**Table 1:** Mean model outputs from 10 iterations of MaxEnt models to predict habitat suitability in California for four *Amblyomma* tick species.

Environmental Predictor Variable	*A. americanum*		*A. maculatum*		*A. cajennense*		*A. mixtum*	
	Percent Contribution	Permutation Importance	Percent Contribution	Permutation Importance	Percent Contribution	Permutation Importance	Percent Contribution	Permutation Importance
BIO1 = Annual mean temperature	na	na	na	na	68.2	25.8	na	na
BIO2 = Mean diurnal range (mean of monthly (max temp - min temp))	12.8	33.3	na	na	na	na	na	na
BIO3 = Isothermality (BIO2/BIO7) (* 100)	na	na	na	na	na	na	na	na
BIO4 = Temperature seasonality (standard deviation *100)	na	na	na	na	na	na	na	na
BIO5 = Max temperature of warmest month	na	na	na	na	na	na	na	na
BIO6 = Min temperature of coldest month	27.3	38.4	38.4	40.5	8.8	25.8	79.6	67.9
BIO7 = Temperature annual range (BIO5 - BIO6)	na	na	na	na	na	na	na	na
BIO8 = Mean temperature of wettest quarter	na	na	na	na	na	na	14.3	12.3
BIO9 = Mean temperature of driest quarter	na	na	na	na	na	na	na	na
BIO10 = Mean temperature of warmest quarter	na	na	na	na	na	na	na	na
BIO11 = Mean temperature of coldest quarter	na	na	na	na	na	na	na	na
BIO12 = Annual precipitation	na	na	na	na	na	na	na	na
BIO13 = Precipitation of wettest month	na	na	18.9	15.2	na	na	na	na
BIO14 = Precipitation of driest month	59.9	28.3	na	na	na	na	na	na
BIO15 = Precipitation seasonality (coefficient of variation)	na	na	na	na	12.4	21.9	na	na
BIO16 = Precipitation of wettest quarter	na	na	na	na	na	na	na	na
BIO17 = Precipitation of driest quarter	na	na	na	na	na	na	na	na
BIO18 = Precipitation of warmest quarter	na	na	na	na	na	na	na	na
BIO19 = Precipitation of coldest quarter	na	na	na	na	na	na	na	na
Elevation	na	na	30.6	29.8	na	na	0.7	1.0
Slope	na	na	na	na	na	na	5.3	18.8
NDVI average (1985-2000)	na	na	12.2	14.5	3.3	12.8	na	na
NDVI standard deviation (1985-2000)	na	na	na	na	7.4	13.7	na	na
Average test AUC from 10 iterations (range)	0.78 (0.69–0.84)		0.74 (0.66–0.84)		0.64 (0.55–0.75)		0.73 (0.56–0.94)	
Maximum sensitivity at maximum specificity	0.24		0.30		0.61		0.61	

NDVI: normalized difference vegetation index, AUC: average area under the curve (of receiver operator characteristics), na: Variable not included in final model due to collinearity with other variables that were considered more biologically important and/or contributed more to the model.

## Supplementary Materials

The following are available online at https://www.mdpi.com/2075-4450/10/7/201/s1.
