# Supplementary material for: Modeling Potential Habitat for Amblyomma Tick Species in California
Source: Insects. 2019 Jul 8;10(7):201. doi: 10.3390/insects10070201 (PMC6681388; doi:10.3390/insects10070201)
Supplement: Supplementary file 1 [file insects-10-00201-s001.pdf]

Table S1. Each geolocation recorded for ticks in the species *Amblyomma americanum* in the literature and publicly available online databases.

Latitude	Longitude	Year	Data Source	Reference
46.25	-114.17	1966		Australian Museum
28.31	-82.46	2011		BISON
28.51	-81.32	2009		BISON
29.68	-82.36	2006		BISON
29.68	-82.36	2006		BISON
29.99	-100.31	2009		BISON
29.99	-81.86	2014		BISON
29.99	-81.86	2014		BISON
30.24	-97.69	2005		BISON
30.46	-84.28	2017		BISON
32.60	-85.35	2008		BISON
32.60	-85.35	2008		BISON
32.80	-79.94	2012		BISON
33.42	-88.88	2016		BISON
33.55	-86.90	2013		BISON
33.70	-84.77	2011		BISON
33.95	-83.37	2016		BISON
34.72	-96.69	2013		BISON
34.72	-96.69	2013		BISON
34.72	-96.69	2015		BISON
35.05	-78.83	2004		BISON
35.05	-78.83	2004		BISON
35.05	-78.83	2004	BISON	BISON
35.05	-78.83	2005		BISON
35.05	-78.83	2006		BISON
35.21	-97.32	2009		BISON
35.21	-97.32	2009		BISON
35.79	-78.65	2011		BISON
35.79	-78.65	2012		BISON
35.79	-78.65	2011		BISON
35.91	-93.22	2009		BISON
35.97	-77.99	2009		BISON
35.99	-83.94	2015		BISON
36.08	-79.79	2011		BISON
36.34	-94.26	2015		BISON
36.62	-96.41	2007		BISON
36.62	-96.41	2008		BISON
36.62	-96.41	2008		BISON
36.62	-96.41	2008		BISON
36.62	-96.41	2008		BISON
36.62	-96.41	2008		BISON
36.62	-96.41	2008		BISON
36.62	-96.41	2009		BISON
36.62	-96.41	2009		BISON
36.62	-96.41	2009		BISON

36.62	-96.41	2009	BISON
36.62	-96.41	2009	BISON
36.62	-96.41	2011	BISON
36.62	-96.41	2012	BISON
36.62	-96.41	2012	BISON
37.36	-77.05	2014	BISON
37.62	-84.87	2016	BISON
37.83	-78.28	2011	BISON
37.97	-85.70	2016	BISON
37.97	-85.70	2016	BISON
38.22	-75.31	2014	BISON
38.43	-88.43	2007	BISON
38.68	-75.34	2012	BISON
38.83	-76.85	2017	BISON
38.88	-94.82	2009	BISON
38.99	-76.56	2015	BISON
38.99	-76.56	2015	BISON
39.01	-94.34	2015	BISON
39.16	-86.52	2011	BISON
39.20	-84.54	2013	BISON
39.58	-75.64	2013	BISON
39.80	-74.96	2011	BISON
39.80	-74.96	2012	BISON
39.80	-74.96	2012	BISON
39.80	-74.96	2016	BISON
39.88	-74.66	2008	BISON
39.88	-74.66	2009	BISON
39.88	-74.66	2013	BISON
39.97	-75.75	2014	BISON
40.75	-87.83	2015	BISON
40.94	-72.69	2006	BISON
40.94	-72.69	2009	BISON
40.94	-72.69	2009	BISON
40.94	-72.69	2009	BISON
41.40	-71.62	2012	BISON
42.48	-71.40	2014	BISON
43.43	-70.67	2013	BISON
44.65	-71.29	2006	BISON
27.45	-80.33	Not specified	Illinois Natural History Survey
28.00	-80.55	Not specified	Illinois Natural History Survey
28.02	-81.73	Not specified	Illinois Natural History Survey
28.87	-81.27	Not specified	Illinois Natural History Survey
31.14	-99.33	Not specified	Illinois Natural History Survey
34.50	-106.00	Not specified	Illinois Natural History Survey
34.50	-93.06	1952	Illinois Natural History Survey
36.26	-90.97	1962	Illinois Natural History Survey
36.32	-91.48	1962	Illinois Natural History Survey
36.33	-94.12	Not specified	Illinois Natural History Survey
36.33	-94.12	Not specified	Illinois Natural History Survey
37.25	-90.53	Not specified	Illinois Natural History Survey

37.38	-88.66	1965		Illinois Natural History Survey
37.69	-88.92	Not specified		Illinois Natural History Survey
37.76	-89.08	1966		Illinois Natural History Survey
37.82	-90.24	1958		Illinois Natural History Survey
38.43	-89.48	1988		Illinois Natural History Survey
39.58	-88.65	1999		Illinois Natural History Survey
40.11	-88.21	1957		Illinois Natural History Survey
40.11	-88.21	1957		Illinois Natural History Survey
42.28	-88.44	1956		Illinois Natural History Survey
46.25	-114.16	Not specified		Illinois Natural History Survey
30.53	-84.29	2016		iNaturalist Research-grade Observation
32.76	-97.68	2016		iNaturalist Research-grade Observation
32.84	-97.46	2016		iNaturalist Research-grade Observation
33.33	-87.79	2016		iNaturalist Research-grade Observation
33.38	-84.61	2014		iNaturalist Research-grade Observation
34.21	-96.64	2016		iNaturalist Research-grade Observation
35.16	-90.71	2016		iNaturalist Research-grade Observation
35.16	-90.71	2016		iNaturalist Research-grade Observation
35.36	-76.97	2016		iNaturalist Research-grade Observation
35.51	-93.32	2015		iNaturalist Research-grade Observation
36.10	-86.53	2016		iNaturalist Research-grade Observation
36.15	-97.08	2012		iNaturalist Research-grade Observation
37.27	-89.04	2016		iNaturalist Research-grade Observation
37.33	-77.21	2014		iNaturalist Research-grade Observation
37.37	-88.95	2016		iNaturalist Research-grade Observation
37.37	-88.93	2016		iNaturalist Research-grade Observation
37.41	-77.70	2013		iNaturalist Research-grade Observation
37.49	-89.35	2016		iNaturalist Research-grade Observation
37.72	-89.25	2016		iNaturalist Research-grade Observation
38.55	-90.42	2016		iNaturalist Research-grade Observation
38.64	-77.23	2014		iNaturalist Research-grade Observation
38.75	-77.12	2012		iNaturalist Research-grade Observation
38.82	-77.51	2016		iNaturalist Research-grade Observation
38.95	-92.45	2016		iNaturalist Research-grade Observation
38.96	-74.96	2016		iNaturalist Research-grade Observation
39.96	-74.14	2016		iNaturalist Research-grade Observation
28.31	-82.46	2011		BISON
28.51	-81.32	2009		BISON
29.68	-82.36	2006		BISON
29.68	-82.36	2006		BISON
29.99	-100.31	2009		BISON
29.99	-81.86	2014		BISON
29.99	-81.86	2014		BISON
30.24	-97.69	2005	GBIF*	BISON
30.46	-84.28	2017		BISON
32.60	-85.35	2008		BISON
32.60	-85.35	2008		BISON
32.80	-79.94	2012		BISON
33.42	-88.88	2016		BISON
33.55	-86.90	2013		BISON

33.70	-84.77	2011	BISON
33.95	-83.37	2016	BISON
34.72	-96.69	2015	BISON
34.72	-96.69	2013	BISON
34.72	-96.69	2013	BISON
35.05	-78.83	2006	BISON
35.05	-78.83	2004	BISON
35.05	-78.83	2004	BISON
35.05	-78.83	2004	BISON
35.05	-78.83	2005	BISON
35.21	-97.32	2009	BISON
35.21	-97.32	2009	BISON
35.79	-78.65	2011	BISON
35.79	-78.65	2011	BISON
35.79	-78.65	2012	BISON
35.91	-93.22	2009	BISON
35.97	-77.99	2009	BISON
35.99	-83.94	2015	BISON
36.08	-79.79	2011	BISON
36.34	-94.26	2015	BISON
36.62	-96.41	2012	BISON
36.62	-96.41	2008	BISON
36.62	-96.41	2009	BISON
36.62	-96.41	2011	BISON
36.62	-96.41	2009	BISON
36.62	-96.41	2008	BISON
36.62	-96.41	2008	BISON
36.62	-96.41	2012	BISON
36.62	-96.41	2008	BISON
36.62	-96.41	2008	BISON
36.62	-96.41	2009	BISON
36.62	-96.41	2009	BISON
36.62	-96.41	2009	BISON
36.62	-96.41	2008	BISON
36.62	-96.41	2007	BISON
37.36	-77.05	2014	BISON
37.62	-84.87	2016	BISON
37.83	-78.28	2011	BISON
37.97	-85.70	2016	BISON
37.97	-85.70	2016	BISON
38.22	-75.31	2014	BISON
38.43	-88.43	2007	BISON
38.68	-75.34	2012	BISON
38.83	-76.85	2017	BISON
38.88	-94.82	2009	BISON
38.99	-76.56	2015	BISON
38.99	-76.56	2015	BISON
39.01	-94.34	2015	BISON
39.16	-86.52	2011	BISON
39.20	-84.54	2013	BISON

39.58	-75.64	2013	BISON
39.80	-74.96	2012	BISON
39.80	-74.96	2011	BISON
39.80	-74.96	2016	BISON
39.80	-74.96	2012	BISON
39.88	-74.66	2013	BISON
39.88	-74.66	2008	BISON
39.88	-74.66	2009	BISON
39.97	-75.75	2014	BISON
40.75	-87.83	2015	BISON
40.94	-72.69	2009	BISON
40.94	-72.69	2009	BISON
40.94	-72.69	2009	BISON
40.94	-72.69	2006	BISON
41.40	-71.62	2012	BISON
42.48	-71.40	2014	BISON
43.43	-70.67	2013	BISON
44.65	-71.29	2006	BISON
38.09	-88.95	1984	Canadian Museum of Nature
34.68	-98.40	2003	Denver Museum of Nature & Science
38.03	-78.50	Not specified	Essig Museum of Entomology Research
27.45	-80.33	Not specified	Illinois Natural History Survey
28.02	-81.73	Not specified	Illinois Natural History Survey
28.87	-81.27	Not specified	Illinois Natural History Survey
31.14	-99.33	Not specified	Illinois Natural History Survey
34.50	-106.00	Not specified	Illinois Natural History Survey
34.50	-93.06	1952	Illinois Natural History Survey
36.26	-90.97	1962	Illinois Natural History Survey
36.32	-91.48	1962	Illinois Natural History Survey
36.33	-94.12	Not specified	Illinois Natural History Survey
36.33	-94.12	Not specified	Illinois Natural History Survey
37.25	-90.53	Not specified	Illinois Natural History Survey
37.38	-88.66	1965	Illinois Natural History Survey
37.69	-88.92	Not specified	Illinois Natural History Survey
37.76	-89.08	1966	Illinois Natural History Survey
37.82	-90.24	1958	Illinois Natural History Survey
38.43	-89.48	1988	Illinois Natural History Survey
39.58	-88.65	1999	Illinois Natural History Survey
40.11	-88.21	1957	Illinois Natural History Survey
40.11	-88.21	1957	Illinois Natural History Survey
42.28	-88.44	1956	Illinois Natural History Survey
46.25	-114.16	Not specified	Illinois Natural History Survey
27.62	-97.22	2018	iNaturalist Research-grade Observation
28.57	-81.00	2018	iNaturalist Research-grade Observation
29.62	-98.68	2018	iNaturalist Research-grade Observation
29.63	-82.37	2018	iNaturalist Research-grade Observation
29.72	-82.22	2018	iNaturalist Research-grade Observation
29.83	-81.98	2017	iNaturalist Research-grade Observation
29.94	-81.98	2018	iNaturalist Research-grade Observation
30.20	-83.93	2017	iNaturalist Research-grade Observation

30.27	-95.68	2017	iNaturalist Research-grade Observation
30.44	-84.18	2018	iNaturalist Research-grade Observation
30.53	-84.29	2016	iNaturalist Research-grade Observation
30.72	-94.23	2016	iNaturalist Research-grade Observation
30.93	-84.15	2017	iNaturalist Research-grade Observation
31.59	-95.37	2017	iNaturalist Research-grade Observation
32.55	-85.03	2018	iNaturalist Research-grade Observation
32.55	-85.03	2018	iNaturalist Research-grade Observation
32.68	-80.35	2017	iNaturalist Research-grade Observation
32.68	-80.35	2017	iNaturalist Research-grade Observation
32.72	-97.12	2017	iNaturalist Research-grade Observation
32.76	-97.68	2016	iNaturalist Research-grade Observation
32.76	-97.68	2015	iNaturalist Research-grade Observation
32.79	-97.41	2018	iNaturalist Research-grade Observation
32.84	-97.46	2016	iNaturalist Research-grade Observation
32.86	-97.48	2018	iNaturalist Research-grade Observation
32.94	-97.48	2013	iNaturalist Research-grade Observation
33.19	-87.48	2018	iNaturalist Research-grade Observation
33.24	-87.56	2017	iNaturalist Research-grade Observation
33.35	-93.18	2018	iNaturalist Research-grade Observation
33.38	-84.61	2014	iNaturalist Research-grade Observation
33.78	-83.37	2017	iNaturalist Research-grade Observation
33.79	-95.63	2017	iNaturalist Research-grade Observation
33.79	-95.67	2017	iNaturalist Research-grade Observation
33.81	-95.68	2017	iNaturalist Research-grade Observation
34.21	-96.64	2016	iNaturalist Research-grade Observation
34.31	-94.63	2018	iNaturalist Research-grade Observation
34.38	-85.63	2017	iNaturalist Research-grade Observation
34.38	-85.63	2017	iNaturalist Research-grade Observation
34.76	-92.31	2018	iNaturalist Research-grade Observation
34.98	-85.81	2018	iNaturalist Research-grade Observation
35.00	-85.37	2018	iNaturalist Research-grade Observation
35.01	-85.36	2018	iNaturalist Research-grade Observation
35.08	-85.59	2017	iNaturalist Research-grade Observation
35.16	-85.12	2018	iNaturalist Research-grade Observation
35.16	-90.71	2016	iNaturalist Research-grade Observation
35.16	-90.71	2016	iNaturalist Research-grade Observation
35.17	-85.12	2015	iNaturalist Research-grade Observation
35.21	-97.25	2017	iNaturalist Research-grade Observation
35.21	-97.23	2017	iNaturalist Research-grade Observation
35.22	-97.21	2018	iNaturalist Research-grade Observation
35.27	-75.53	2017	iNaturalist Research-grade Observation
35.35	-82.78	2018	iNaturalist Research-grade Observation
35.36	-76.97	2016	iNaturalist Research-grade Observation
35.53	-78.80	2018	iNaturalist Research-grade Observation
35.62	-78.93	2017	iNaturalist Research-grade Observation
35.69	-94.84	2018	iNaturalist Research-grade Observation
35.71	-77.95	2017	iNaturalist Research-grade Observation
35.75	-78.72	2017	iNaturalist Research-grade Observation
35.81	-78.48	2018	iNaturalist Research-grade Observation

35.88	-84.30	2016	iNaturalist Research-grade Observation
35.88	-84.30	2016	iNaturalist Research-grade Observation
35.89	-78.96	2016	iNaturalist Research-grade Observation
35.92	-78.39	2018	iNaturalist Research-grade Observation
35.93	-78.77	2018	iNaturalist Research-grade Observation
35.97	-78.97	2018	iNaturalist Research-grade Observation
35.97	-78.97	2017	iNaturalist Research-grade Observation
35.97	-78.96	2018	iNaturalist Research-grade Observation
35.97	-78.96	2018	iNaturalist Research-grade Observation
35.97	-78.96	2018	iNaturalist Research-grade Observation
36.00	-78.93	2017	iNaturalist Research-grade Observation
36.07	-94.21	2018	iNaturalist Research-grade Observation
36.10	-86.53	2016	iNaturalist Research-grade Observation
36.15	-97.08	2012	iNaturalist Research-grade Observation
36.22	-95.91	2017	iNaturalist Research-grade Observation
36.29	-95.65	2018	iNaturalist Research-grade Observation
36.30	-80.06	2018	iNaturalist Research-grade Observation
36.55	-93.78	2017	iNaturalist Research-grade Observation
36.65	-91.78	2018	iNaturalist Research-grade Observation
36.70	-78.67	2018	iNaturalist Research-grade Observation
36.85	-89.89	2017	iNaturalist Research-grade Observation
36.90	-87.63	2018	iNaturalist Research-grade Observation
36.97	-94.36	2016	iNaturalist Research-grade Observation
37.03	-91.17	2018	iNaturalist Research-grade Observation
37.04	-78.46	2018	iNaturalist Research-grade Observation
37.04	-78.28	2018	iNaturalist Research-grade Observation
37.06	-76.31	2018	iNaturalist Research-grade Observation
37.07	-76.31	2018	iNaturalist Research-grade Observation
37.17	-91.04	2017	iNaturalist Research-grade Observation
37.19	-90.99	2017	iNaturalist Research-grade Observation
37.20	-78.45	2018	iNaturalist Research-grade Observation
37.33	-77.21	2014	iNaturalist Research-grade Observation
37.37	-88.93	2016	iNaturalist Research-grade Observation
37.38	-79.14	2017	iNaturalist Research-grade Observation
37.39	-86.23	2018	iNaturalist Research-grade Observation
37.41	-76.94	2018	iNaturalist Research-grade Observation
37.41	-76.71	2018	iNaturalist Research-grade Observation
37.41	-77.70	2013	iNaturalist Research-grade Observation
37.45	-77.64	2017	iNaturalist Research-grade Observation
37.45	-77.64	2017	iNaturalist Research-grade Observation
37.49	-89.35	2016	iNaturalist Research-grade Observation
37.54	-84.90	2016	iNaturalist Research-grade Observation
37.56	-77.47	2014	iNaturalist Research-grade Observation
37.56	-93.41	2015	iNaturalist Research-grade Observation
37.59	-88.38	2017	iNaturalist Research-grade Observation
37.65	-97.45	2018	iNaturalist Research-grade Observation
37.72	-89.20	2016	iNaturalist Research-grade Observation
37.72	-89.25	2016	iNaturalist Research-grade Observation
37.74	-97.26	2017	iNaturalist Research-grade Observation
38.01	-77.92	2018	iNaturalist Research-grade Observation

38.02	-77.92	2017	iNaturalist Research-grade Observation
38.17	-86.32	2017	iNaturalist Research-grade Observation
38.21	-75.80	2016	iNaturalist Research-grade Observation
38.21	-75.80	2016	iNaturalist Research-grade Observation
38.55	-90.42	2016	iNaturalist Research-grade Observation
38.58	-82.79	2017	iNaturalist Research-grade Observation
38.59	-92.23	2017	iNaturalist Research-grade Observation
38.59	-92.23	2018	iNaturalist Research-grade Observation
38.59	-92.23	2018	iNaturalist Research-grade Observation
38.62	-77.53	2018	iNaturalist Research-grade Observation
38.63	-77.54	2017	iNaturalist Research-grade Observation
38.64	-77.23	2018	iNaturalist Research-grade Observation
38.64	-77.23	2014	iNaturalist Research-grade Observation
38.68	-77.25	2013	iNaturalist Research-grade Observation
38.75	-77.12	2012	iNaturalist Research-grade Observation
38.76	-75.73	2017	iNaturalist Research-grade Observation
38.77	-75.09	2014	iNaturalist Research-grade Observation
38.79	-76.70	2018	iNaturalist Research-grade Observation
38.79	-93.22	2018	iNaturalist Research-grade Observation
38.81	-77.52	2014	iNaturalist Research-grade Observation
38.95	-92.45	2018	iNaturalist Research-grade Observation
38.95	-92.45	2018	iNaturalist Research-grade Observation
38.95	-92.45	2017	iNaturalist Research-grade Observation
38.95	-92.45	2018	iNaturalist Research-grade Observation
38.95	-92.45	2017	iNaturalist Research-grade Observation
38.95	-92.45	2017	iNaturalist Research-grade Observation
38.95	-92.45	2017	iNaturalist Research-grade Observation
38.95	-92.45	2017	iNaturalist Research-grade Observation
38.95	-92.45	2016	iNaturalist Research-grade Observation
38.96	-77.33	2015	iNaturalist Research-grade Observation
38.96	-74.96	2016	iNaturalist Research-grade Observation
38.97	-90.51	2017	iNaturalist Research-grade Observation
38.98	-77.24	2013	iNaturalist Research-grade Observation
38.98	-77.24	2013	iNaturalist Research-grade Observation
39.00	-76.82	2018	iNaturalist Research-grade Observation
39.06	-76.79	2016	iNaturalist Research-grade Observation
39.13	-77.15	2018	iNaturalist Research-grade Observation
39.18	-82.40	2017	iNaturalist Research-grade Observation
39.19	-96.55	2018	iNaturalist Research-grade Observation
39.20	-96.68	2018	iNaturalist Research-grade Observation
39.22	-76.80	2018	iNaturalist Research-grade Observation
39.24	-76.60	2016	iNaturalist Research-grade Observation
39.32	-76.51	2018	iNaturalist Research-grade Observation
39.41	-94.26	2018	iNaturalist Research-grade Observation
39.51	-75.15	2018	iNaturalist Research-grade Observation
39.77	-74.37	2016	iNaturalist Research-grade Observation
39.96	-74.14	2016	iNaturalist Research-grade Observation
40.00	-74.82	2018	iNaturalist Research-grade Observation
40.27	-74.65	2018	iNaturalist Research-grade Observation
40.47	-79.95	2018	iNaturalist Research-grade Observation

40.47	-74.42	2018		iNaturalist Research-grade Observation
40.47	-74.42	2017		iNaturalist Research-grade Observation
40.54	-74.23	2017		iNaturalist Research-grade Observation
40.54	-74.23	2017		iNaturalist Research-grade Observation
40.90	-72.81	2018		iNaturalist Research-grade Observation
40.90	-72.81	2018		iNaturalist Research-grade Observation
40.99	-87.57	2015		iNaturalist Research-grade Observation
41.29	-96.10	2018		iNaturalist Research-grade Observation
41.98	-87.74	2015		iNaturalist Research-grade Observation
42.17	-80.07	2018		iNaturalist Research-grade Observation
42.24	-87.82	2018		iNaturalist Research-grade Observation
43.42	-91.01	2018		iNaturalist Research-grade Observation
30.32	-98.95	Not specified		Ohio State University Acarology Laboratory
33.70	-84.35	Not specified		Ohio State University Acarology Laboratory
33.70	-84.35	Not specified		Ohio State University Acarology Laboratory
33.70	-84.35	Not specified		Ohio State University Acarology Laboratory
34.23	-91.90	1954		Ohio State University Acarology Laboratory
34.23	-91.90	1954		Ohio State University Acarology Laboratory
34.23	-91.90	1954		Ohio State University Acarology Laboratory
34.23	-91.90	1954		Ohio State University Acarology Laboratory
34.23	-91.90	1954		Ohio State University Acarology Laboratory
35.97	-94.23	1955		Ohio State University Acarology Laboratory
39.05	-76.80	1955		Ohio State University Acarology Laboratory
40.94	-74.40	Not specified		Ohio State University Acarology Laboratory
42.41	-83.93	1951		Ohio State University Acarology Laboratory
42.41	-83.93	1951		Ohio State University Acarology Laboratory
42.41	-83.93	1951		Ohio State University Acarology Laboratory
42.41	-83.93	1951		Ohio State University Acarology Laboratory
42.41	-83.93	1951		Ohio State University Acarology Laboratory
42.41	-83.93	1951		Ohio State University Acarology Laboratory
42.41	-83.93	1951		Ohio State University Acarology Laboratory
42.41	-83.93	1951		Ohio State University Acarology Laboratory
42.41	-83.93	1951		Ohio State University Acarology Laboratory
42.41	-83.93	1951		Ohio State University Acarology Laboratory
46.25	-114.17	1966		South African National Biodiversity Institute
36.69	-92.42	2015		
36.75	-91.32	2015		
36.83	-92.10	2015		[1]
36.87	-92.47	2015		
37.19	-91.45	2015		
37.41	-92.33	2015		
37.04	-91.19	2006		[2]
37.08	-91.06	2006	Literature	
36.94	-90.35	2001		[3]
35.61	-79.16	2009		[4]
35.75	-79.06	2009		
40.18	-92.60	2007-2010		[5]
39.06	-76.82	2006		[6]
39.08	-76.44	1998-2007		[7]
31.61	-91.23	2008		[8]

33.27	-88.78	2008	
33.45	-88.59	2008	
34.23	-88.63	2008	
34.60	-88.19	2008	
34.66	-89.46	2008	
35.13	-79.20	2011	[9]
30.12	-85.72	1994-1996	[10]
39.11	-96.61	1995-1996	[11]
33.35	-83.15	1987-1991	[12]
32.00	-85.46	1988-1990	[13]
32.91	-86.35	1988-1990	
32.96	-87.46	1988-1990	
33.75	-85.62	1988-1990	
39.26	-75.47	2013	[14]
40.66	-73.08	1986-1987	[15]
31.84	-90.70	1999-2000	[16]
34.67	-89.46	1999-2000	
39.83	-77.25	2009	[17]
36.13	-97.22	2008	[18]
38.85	-77.40	2011	[19]
38.85	-77.40	2011	
38.85	-77.40	2011	
40.05	-92.84	2014	[20]
40.05	-92.84	2014	
40.05	-92.84	2014	
40.05	-92.84	2014	
40.12	-92.64	2014	
40.12	-92.64	2014	
40.12	-92.64	2014	
40.12	-92.64	2014	
40.16	-92.62	2014	
40.16	-92.62	2014	
40.16	-92.62	2014	
40.16	-92.62	2014	
40.29	-92.80	2014	
40.29	-92.80	2014	
40.29	-92.80	2014	
40.29	-92.80	2014	
29.95	-81.95	2011	[21]
41.61	-71.32	1992	[22]
33.26	-88.84	1992-1993	[23]
38.07	-77.32	2005	[24]
38.52	-90.54	2009	[25]
31.84	-81.10	2005-2009	[26]
31.84	-81.10	2005-2009	
33.05	-83.77	2005-2009	
33.05	-83.77	2005-2009	
33.46	-83.73	2005-2009	
33.46	-83.73	2005-2009	
33.63	-84.15	2005-2009	

33.63	-84.15	2005-2009	
33.63	-84.15	2005-2009	
35.67	-94.84	1971, 1973	[27]
37.68	-89.25	1983-1984	[28]
30.06	-99.51	Not specified	[29]
40.06	-96.00	2012	
40.18	-96.07	2012	
40.25	-95.53	2012	[30]
40.76	-96.72	2012	
40.84	-96.57	2012	
41.03	-96.25	2012	
35.12	-89.22	2012-2014	
35.12	-89.22	2012-2014	[31,32]
35.12	-89.22	2012-2014	
35.12	-89.22	2012-2014	
30.86	-81.44	2002-2005	
31.07	-81.42	2002-2005	
31.62	-81.17	2002-2005	
31.88	-81.18	2002-2005	[33]
33.10	-83.71	2002-2005	
33.39	-83.49	2002-2005	
33.67	-83.59	2002-2005	
34.18	-83.14	2002-2005	
32.50	-81.21	Not specified	
32.50	-81.21	Not specified	
32.50	-81.21	Not specified	
32.50	-81.21	Not specified	
32.50	-81.21	Not specified	
32.50	-81.21	Not specified	
32.50	-81.21	Not specified	
32.50	-81.21	Not specified	
32.50	-81.21	Not specified	
32.50	-81.21	Not specified	
32.50	-81.21	Not specified	
32.50	-81.21	Not specified	
32.50	-81.21	Not specified	
32.50	-81.21	Not specified	[34]
32.50	-81.21	Not specified	
32.50	-81.21	Not specified	
32.50	-81.21	Not specified	
32.50	-81.21	Not specified	
40.91	-72.83	Not specified	
40.91	-72.83	Not specified	
40.94	-72.93	Not specified	
40.94	-72.93	Not specified	
40.94	-72.93	Not specified	
40.94	-72.93	Not specified	
40.94	-72.93	Not specified	
40.94	-72.93	Not specified	
40.94	-72.93	Not specified	

40.94	-72.93	Not specified	
40.94	-72.93	Not specified	
40.94	-72.93	Not specified	
40.94	-72.93	Not specified	
40.94	-72.93	Not specified	
40.94	-72.93	Not specified	
40.94	-72.93	Not specified	
40.94	-72.93	Not specified	
40.94	-72.93	Not specified	
40.94	-72.93	Not specified	
40.94	-72.93	Not specified	
40.94	-72.93	Not specified	
40.94	-72.93	Not specified	
40.94	-72.93	Not specified	
40.94	-72.93	Not specified	
40.96	-72.80	Not specified	
40.96	-72.80	Not specified	
35.24	-97.32	2015	
35.24	-97.32	2015	
35.42	-99.06	2015	
35.93	-98.43	2015	
36.06	-97.40	2015	[35]
36.06	-97.40	2015	
36.06	-97.40	2015	
36.13	-97.07	2015	
36.16	-97.20	2015	
35.48	-97.46	2015	
35.48	-97.46	2015	
35.49	-97.51	2015	
35.49	-97.51	2015	
35.51	-97.52	2015	
35.51	-97.52	2015	
35.52	-97.55	2015	
35.52	-97.55	2015	
35.53	-97.61	2015	
35.53	-97.61	2015	[36]
35.55	-97.57	2015	
35.55	-97.57	2015	
35.55	-97.58	2015	
35.55	-97.58	2015	
35.56	-97.61	2015	
35.56	-97.61	2015	
35.59	-97.61	2015	
35.59	-97.61	2015	
35.61	-97.61	2015	
35.61	-97.61	2015	
38.71	-77.24	2010-2011	[37]
38.85	-77.48	2010-2011	
34.59	-77.33	1990-1993	[38]
40.18	-92.60	2007	[39]

36.06	-97.23	2006-2007, 2009-2010	[40]
35.11	-89.21	2013	
35.11	-89.21	2013	
35.41	-86.81	2013	
35.41	-86.81	2013	
35.72	-86.95	2013	
35.72	-86.95	2013	
35.82	-83.82	2013	[41]
35.82	-83.82	2013	
35.84	-85.06	2013	
35.84	-85.06	2013	
36.11	-82.84	2013	
36.11	-82.84	2013	
36.47	-86.84	2013	
36.47	-86.84	2013	
35.72	-79.45	Not specified	[42]
29.60	-99.74	1990-1992	
30.62	-95.52	1990-1992	
31.04	-98.48	1990-1992	
31.86	-99.03	1990-1992	[43]
31.91	-95.90	1990-1992	
32.26	-97.55	1990-1992	
32.48	-95.30	1990-1992	
32.86	-98.48	1990-1992	
31.15	-97.82	2008-2011	
31.15	-97.82	2008-2011	
31.15	-97.82	2008-2011	
31.15	-97.82	2008-2011	
31.15	-97.82	2008-2011	[44]
31.15	-97.82	2008-2011	
31.15	-97.82	2008-2011	
31.15	-97.82	2008-2011	
31.15	-97.82	2008-2011	
31.15	-97.82	2008-2011	
38.07	-77.33	1987	[45]
39.86	-94.79	2013	
39.86	-94.79	2013	
39.86	-94.79	2013	
39.92	-94.89	2013	
39.92	-94.89	2013	
39.92	-94.89	2013	
39.96	-94.97	2013	[46]
39.96	-94.97	2013	
39.96	-94.97	2013	
39.96	-94.98	2013	
39.96	-94.98	2013	
39.96	-94.98	2013	
40.16	-94.54	2013	
40.16	-94.54	2013	

40.16	-94.54	2013	
40.57	-94.67	2013	
40.57	-94.67	2013	
40.57	-94.67	2013	
29.48	-82.98	2010-2011	
29.48	-82.98	2010-2011	
29.50	-82.97	2010-2011	
29.59	-82.93	2010-2011	
29.71	-82.46	2010-2011	
29.71	-82.46	2010-2011	
29.71	-82.46	2010-2011	
29.71	-82.46	2010-2011	[47]
29.91	-82.57	2010-2011	
29.91	-82.57	2010-2011	
29.91	-82.57	2010-2011	
29.92	-82.59	2010-2011	
29.96	-82.78	2010-2011	
30.46	-81.42	2010-2011	
29.48	-82.97	2010-2012	
29.48	-82.97	2010-2012	
29.59	-82.94	2010-2012	
29.74	-82.43	2010-2012	
29.74	-82.43	2010-2012	[48]
29.74	-82.43	2010-2012	
29.92	-82.57	2010-2012	
29.92	-82.57	2010-2012	
29.92	-82.57	2010-2012	
40.26	-74.11	2003-2004	[49]
40.26	-74.11	2003-2004	
40.16	-74.13	2004	
40.16	-74.13	2004	
40.17	-74.18	2004	
40.17	-74.18	2004	
40.17	-74.21	2004	
40.17	-74.21	2004	
40.21	-74.09	2004	
40.21	-74.09	2004	
40.26	-74.25	2004	[50]
40.26	-74.25	2004	
40.27	-74.16	2004	
40.27	-74.32	2004	
40.27	-74.32	2004	
40.28	-74.09	2004	
40.28	-74.09	2004	
40.30	-74.14	2004	
40.30	-74.14	2004	
40.15	-74.09	2008	
40.16	-74.51	2008	[51]
40.17	-74.19	2008	
40.21	-74.05	2008	

40.22	-74.44	2008	
40.25	-74.04	2008	
40.29	-74.35	2008	
40.29	-74.10	2008	
40.29	-74.14	2008	
40.30	-74.04	2008	
40.33	-74.27	2008	
40.34	-74.04	2008	
40.38	-74.18	2008	
40.39	-74.09	2008	
40.41	-74.03	2008	
35.70	-94.82	1969	[52]
27.52	-97.87	1996-1997	[53]
28.33	-98.83	1996-1997	
37.07	-77.97	1998-1999	[54]
39.46	-76.12	Not specified	[55]
40.71	-73.16	2013	[56]
37.52	-76.82	2015-2016	
40.72	-73.17	2015-2016	
40.72	-72.90	2015-2016	
40.77	-73.13	2015-2016	[57]
40.80	-73.42	2015-2016	
40.86	-72.75	2015-2016	
40.98	-72.55	2015-2016	
35.12	-89.22	2014	[58]
35.12	-89.22	2012	
35.12	-89.22	2012	
35.12	-89.22	2012	
35.12	-89.22	2012	
35.12	-89.22	2012	
35.12	-89.22	2012	
35.12	-89.22	2012	
35.12	-89.22	2012	
35.12	-89.22	2012	
35.12	-89.22	2012	
35.12	-89.22	2012	
35.12	-89.22	2012	
35.12	-89.22	2012	[59]
35.12	-89.22	2012	
35.12	-89.22	2012	
35.12	-89.22	2012	
35.12	-89.22	2012	
35.12	-89.22	2012	
35.12	-89.22	2012	
35.12	-89.22	2012	
35.12	-89.22	2012	
35.12	-89.22	2012	
35.12	-89.22	2012	
35.12	-89.22	2012	
35.12	-89.22	2012	

35.12	-89.22	2012	
35.12	-89.22	2012	
35.12	-89.22	2012	
35.12	-89.22	2012	
35.12	-89.22	2012	
35.12	-89.22	2012	
35.12	-89.22	2012	
35.12	-89.22	2012	
35.12	-89.22	2012	
35.12	-89.22	2012	
35.12	-89.22	2012	
35.12	-89.22	2012	
35.12	-89.22	2012	
35.12	-89.22	2012	
35.12	-89.22	2012	
35.12	-89.22	2012	
35.12	-89.22	2012	
35.12	-89.22	2012	
35.12	-89.22	2012	
35.12	-89.22	2012	
35.12	-89.22	2012	
35.12	-89.22	2012	
35.12	-89.22	2012	
31.47	-81.24	Not specified	[60]
33.64	-84.17	2010-2011	
33.75	-84.63	2010-2011	[61]
35.26	-75.56	2010-2011	
41.06	-72.31	2010-2011	
33.71	-85.78	2008-2009	
33.72	-85.78	2008-2009	
33.72	-85.79	2008-2009	
33.79	-85.58	2008-2009	
33.79	-85.59	2008-2009	
33.79	-85.59	2008-2009	
33.79	-85.56	2008-2009	
33.79	-85.57	2008-2009	
33.79	-85.57	2008-2009	
33.79	-85.59	2008-2009	[62]
33.79	-85.55	2008-2009	
33.79	-85.56	2008-2009	
33.79	-85.55	2008-2009	
33.79	-85.55	2008-2009	
33.79	-85.55	2008-2009	
33.82	-85.76	2008-2009	
33.83	-85.76	2008-2009	
34.00	-85.45	2008-2009	
34.00	-85.45	2008-2009	
34.01	-85.46	2008-2009	
38.25	-75.16	2008-2009	[63]
38.25	-75.16	2008-2009	

38.25	-75.16	2008-2009	
38.25	-75.16	2008-2009	
38.27	-75.45	2008-2009	
38.27	-75.45	2008-2009	
38.29	-76.52	2008-2009	
38.29	-76.52	2008-2009	
38.35	-75.42	2008-2009	
38.35	-75.42	2008-2009	
38.36	-76.53	2008-2009	
38.36	-76.53	2008-2009	
38.41	-76.44	2008-2009	
38.41	-76.44	2008-2009	
38.41	-76.44	2008-2009	
38.47	-77.02	2008-2009	
38.49	-75.79	2008-2009	
38.49	-75.79	2008-2009	
38.54	-75.93	2008-2009	
38.54	-75.93	2008-2009	
38.54	-75.93	2008-2009	
38.54	-75.93	2008-2009	
38.61	-77.13	2008-2009	
38.61	-77.13	2008-2009	
38.65	-76.81	2008-2009	
38.65	-76.81	2008-2009	
38.65	-76.81	2008-2009	
38.65	-76.81	2008-2009	
38.75	-75.73	2008-2009	
38.75	-75.73	2008-2009	
38.75	-75.73	2008-2009	
38.75	-75.73	2008-2009	
38.96	-75.94	2008-2009	
39.03	-76.41	2008-2009	
39.03	-76.41	2008-2009	
39.04	-77.25	2008-2009	
39.04	-77.25	2008-2009	
39.04	-77.25	2008-2009	
39.04	-77.25	2008-2009	
39.28	-75.77	2008-2009	
39.28	-75.77	2008-2009	
39.31	-77.16	2008-2009	
39.49	-75.98	2008-2009	
39.61	-76.68	2008-2009	
37.66	-89.27	2003-2015	
37.67	-89.32	2003-2015	
37.67	-89.21	2003-2015	
37.67	-89.39	2003-2015	
37.71	-89.25	2003-2015	[64]
37.71	-89.20	2003-2015	
37.72	-89.39	2003-2015	
37.73	-89.32	2003-2015	

37.76	-89.25	2003-2015		
37.76	-89.19	2003-2015		
37.76	-89.31	2003-2015		
37.78	-89.39	2003-2015		
37.79	-89.31	2003-2015		
37.79	-89.25	2003-2015		
36.49	-88.04	1984-1986		[65]
29.49	-82.97	1995-2010		
29.52	-82.29	1995-2010		
29.92	-82.58	1995-2010		
30.14	-85.74	1995-2010		
30.85	-81.46	1995-2010		
31.05	-81.41	1995-2010		
31.59	-91.21	1995-2010		
31.70	-81.28	1995-2010		
31.84	-90.70	1995-2010		
32.37	-80.46	1995-2010		
33.13	-83.75	1995-2010		
33.25	-88.77	1995-2010		
33.40	-83.49	1995-2010		
33.45	-88.59	1995-2010		
33.63	-84.17	1995-2010		
33.65	-88.45	1995-2010		
33.66	-83.61	1995-2010		
33.95	-83.37	1995-2010		
34.14	-78.59	1995-2010		
34.24	-88.62	1995-2010		
34.29	-82.86	1995-2010		
34.44	-89.35	1995-2010	VectorMap	[66–68]
34.61	-88.19	1995-2010		
34.66	-89.47	1995-2010		
34.68	-77.35	1995-2010		
35.26	-75.54	1995-2010		
35.35	-80.09	1995-2010		
35.48	-79.18	1995-2010		
35.90	-75.67	1995-2010		
36.12	-96.12	1995-2010		
37.05	-77.95	1995-2010		
37.06	-91.05	1995-2010		
37.23	-93.00	1995-2010		
38.13	-75.44	1995-2010		
38.23	-75.14	1995-2010		
38.27	-76.50	1995-2010		
38.37	-76.53	1995-2010		
38.40	-76.42	1995-2010		
38.43	-77.25	1995-2010		
38.44	-86.53	1995-2010		
38.47	-77.01	1995-2010		
38.52	-90.55	1995-2010		
38.54	-75.93	1995-2010		

38.62	-77.12	1995-2010
38.64	-76.78	1995-2010
38.72	-75.75	1995-2010
38.97	-75.94	1995-2010
39.02	-76.40	1995-2010
39.03	-76.89	1995-2010
39.07	-95.24	1995-2010
39.28	-75.77	1995-2010
39.42	-76.54	1995-2010
39.48	-75.98	1995-2010
40.18	-92.60	1995-2010
40.22	-74.44	1995-2010
40.29	-74.17	1995-2010
40.65	-73.15	1995-2010
40.87	-72.81	1995-2010
41.04	-71.95	1995-2010
41.07	-72.34	1995-2010
41.60	-71.30	1995-2010

* *A. americanum*: GBIF.org (accessed 24th September 2018) GBIF Occurrence Download https://doi.org/10.15468/dl.z8nzgs

Table S2. Each geolocation recorded for ticks in the species *Amblyomma maculatum* in the literature and publicly available online databases.

Latitude	Longitude	Year	Data Source	Reference
35.05	-78.83	2005	BISON	BugGuide
27.70	-80.57	2006		BugGuide
32.80	-79.94	2007		BugGuide
30.24	-97.69	2013		BugGuide
32.13	-111.78	2015		BugGuide
33.19	-96.58	2017		BugGuide
30.67	-86.59	2007		BugGuide
27.95	-81.69	2007		BugGuide
32.60	-85.35	2008		BugGuide
33.95	-83.37	2008		BugGuide
29.50	-90.04	2009		BugGuide
30.66	-87.75	2009		BugGuide
29.86	-95.39	2010		BugGuide
29.86	-95.39	2010		BugGuide
26.10	-97.48	2010		BugGuide
31.53	-84.21	2011		BugGuide
34.03	-96.77	2012		BugGuide
30.66	-87.75	2012		BugGuide
32.77	-96.78	2013		BugGuide
40.24	-89.91	2015		BugGuide
40.14	-88.20	2015		BugGuide
33.55	-81.63	2015		BugGuide
33.55	-81.63	2015		BugGuide
35.21	-97.32	2017		BugGuide

31.53	-110.85	2017		BugGuide
31.53	-110.85	2017		BugGuide
32.40	-89.12	2017		BugGuide
40.72	-89.60	1990		Illinois Natural History Survey Insect Collection
29.21	-81.02	1945		Illinois Natural History Survey Insect Collection
30.42	-97.76	2013		iNaturalist Research-grade Observation
32.59	-97.10	2014		iNaturalist Research-grade Observation
32.59	-97.10	2014		iNaturalist Research-grade Observation
30.65	-98.04	2010		iNaturalist Research-grade Observation
27.84	-97.07	2016		iNaturalist Research-grade Observation
32.89	-97.28	2016		iNaturalist Research-grade Observation
25.96	-97.25	2015		iNaturalist Research-grade Observation
29.25	-95.60	2016		iNaturalist Research-grade Observation
29.92	-95.22	2016		iNaturalist Research-grade Observation
38.81	-77.53	2016		iNaturalist Research-grade Observation
33.55	-81.63	2015		BugGuide
32.77	-96.78	2013		BugGuide
30.67	-86.59	2007		BugGuide
29.50	-90.04	2009		BugGuide
31.53	-84.21	2011		BugGuide
35.21	-97.32	2017		BugGuide
31.53	-110.85	2017		BugGuide
30.66	-87.75	2012		BugGuide
32.40	-89.12	2017		BugGuide
33.55	-81.63	2015		BugGuide
31.53	-110.85	2017		BugGuide
30.66	-87.75	2009		BugGuide
26.10	-97.48	2010		BugGuide
33.95	-83.37	2008		BugGuide
34.03	-96.77	2012		BugGuide
40.14	-88.20	2015		BugGuide
40.24	-89.91	2015		BugGuide
27.95	-81.69	2007	GBIF*	BugGuide
32.60	-85.35	2008		BugGuide
29.86	-95.39	2010		BugGuide
29.86	-95.39	2010		BugGuide
33.19	-96.58	2017		BugGuide
35.05	-78.83	2005		BugGuide
30.24	-97.69	2013		BugGuide
27.70	-80.57	2006		BugGuide
32.80	-79.94	2007		BugGuide
32.13	-111.78	2015		BugGuide
34.69	-98.45	2004		DMNS
34.70	-98.57	2003		DMNS
34.64	-98.32	2004		DMNS
34.66	-98.28	2004		DMNS
29.21	-81.02	1945		Illinois Natural History Survey
40.72	-89.60	1990		Illinois Natural History Survey
29.94	-95.65	2018		iNaturalist Research-grade Observation
29.20	-94.96	2018		iNaturalist Research-grade Observation

30.52	-84.35	2018	iNaturalist Research-grade Observation
29.20	-94.96	2018	iNaturalist Research-grade Observation
32.88	-97.28	2018	iNaturalist Research-grade Observation
28.66	-97.39	2018	iNaturalist Research-grade Observation
30.61	-88.17	2018	iNaturalist Research-grade Observation
28.65	-97.39	2018	iNaturalist Research-grade Observation
32.77	-97.37	2018	iNaturalist Research-grade Observation
32.97	-93.43	2018	iNaturalist Research-grade Observation
40.40	-89.88	2015	iNaturalist Research-grade Observation
40.13	-88.29	2015	iNaturalist Research-grade Observation
37.80	-97.20	2018	iNaturalist Research-grade Observation
29.73	-84.99	2018	iNaturalist Research-grade Observation
40.86	-73.83	2018	iNaturalist Research-grade Observation
33.73	-90.95	2018	iNaturalist Research-grade Observation
29.63	-82.35	2017	iNaturalist Research-grade Observation
33.71	-97.05	2018	iNaturalist Research-grade Observation
30.12	-97.32	2018	iNaturalist Research-grade Observation
32.73	-97.49	2018	iNaturalist Research-grade Observation
35.44	-97.14	2018	iNaturalist Research-grade Observation
37.02	-94.39	2018	iNaturalist Research-grade Observation
29.37	-94.97	2016	iNaturalist Research-grade Observation
32.72	-96.74	2018	iNaturalist Research-grade Observation
30.62	-92.08	2016	iNaturalist Research-grade Observation
31.00	-92.23	2017	iNaturalist Research-grade Observation
30.66	-104.05	2013	iNaturalist Research-grade Observation
29.90	-95.82	2017	iNaturalist Research-grade Observation
27.67	-97.33	2017	iNaturalist Research-grade Observation
37.47	-77.67	2017	iNaturalist Research-grade Observation
32.57	-80.18	2015	iNaturalist Research-grade Observation
30.39	-97.50	2017	iNaturalist Research-grade Observation
30.45	-88.65	2009	iNaturalist Research-grade Observation
30.31	-95.69	2017	iNaturalist Research-grade Observation
31.15	-97.77	2017	iNaturalist Research-grade Observation
33.18	-86.77	2017	iNaturalist Research-grade Observation
36.84	-96.43	2017	iNaturalist Research-grade Observation
33.70	-95.65	2017	iNaturalist Research-grade Observation
33.79	-95.63	2017	iNaturalist Research-grade Observation
29.22	-94.92	2015	iNaturalist Research-grade Observation
33.81	-95.68	2017	iNaturalist Research-grade Observation
32.57	-96.78	2017	iNaturalist Research-grade Observation
35.81	-78.72	2017	iNaturalist Research-grade Observation
32.57	-96.78	2017	iNaturalist Research-grade Observation
33.13	-97.45	2017	iNaturalist Research-grade Observation
29.11	-95.72	2016	iNaturalist Research-grade Observation
36.15	-97.08	2012	iNaturalist Research-grade Observation
32.89	-97.28	2016	iNaturalist Research-grade Observation
27.84	-97.07	2016	iNaturalist Research-grade Observation
30.65	-98.04	2010	iNaturalist Research-grade Observation
29.25	-95.60	2016	iNaturalist Research-grade Observation
29.92	-95.22	2016	iNaturalist Research-grade Observation

38.81	-77.53	2016		iNaturalist Research-grade Observation
32.59	-97.10	2014		iNaturalist Research-grade Observation
25.96	-97.25	2015		iNaturalist Research-grade Observation
30.42	-97.76	2013		iNaturalist Research-grade Observation
32.59	-97.10	2014		iNaturalist Research-grade Observation
27.48	-81.56	1966		Ohio State University Acarology Laboratory
29.91	-91.66			Ohio State University Acarology Laboratory
29.29	-82.11	1913		Ohio State University Acarology Laboratory
31.19	-89.17	1944		Ohio State University Acarology Laboratory
23.07	-99.12	2000		Texas Natural History Collections
18.63	-103.68	1990		Texas Natural History Collections
31.43	-111.19	2016		
31.43	-111.19	2016		
31.40	-111.19	2016		
31.40	-111.19	2016		
31.41	-111.18	2016		
31.41	-111.18	2016		
31.71	-110.79	2016		
31.71	-110.79	2016		
31.70	-110.79	2016		
31.70	-110.79	2016		[69]
31.86	-110.21	2016		
31.86	-110.21	2016		
31.63	-110.17	2016		
31.63	-110.17	2016		
31.56	-110.14	2016		
31.56	-110.14	2016		
31.38	-110.11	2016		
31.38	-110.11	2016		
31.44	-110.11	2016		
31.44	-110.11	2016	Literature	
29.67	-96.27	2013-2014		[70]
30.12	-85.72	1994-1996		[10]
26.23	-97.35	2015		[71]
26.08	-97.30	2015		
39.26	-75.47	2012		[72]
38.44	-76.10	2013		
39.13	-76.07	2013		[14]
39.26	-75.47	2013		
26.07	-81.41	1984-1990		[73]
25.85	-80.93	1984-1990		
34.39	-89.54	1982		
33.48	-88.84	1982		
33.27	-88.78	1982		[74]
33.30	-88.63	1982		
36.78	-88.05	1982		
31.83	-90.72	1994, 2001-2003		[75]
31.83	-90.72	1994, 2001-2003		

30.42	-88.67	2008-2009	[76]
30.42	-88.67	2008-2009	
38.85	-77.40	2011	
38.85	-77.40	2011	[19]
38.85	-77.40	2011	
38.85	-77.40	2011	
33.27	-88.78	1992-1993	[23]
30.23	-88.02	1998	[77]
31.05	-81.42	1996-1998	
33.48	-88.84	2013-2015	[78]
35.12	-89.22	2012-2014	[31]
35.12	-89.22	2012-2014	
35.12	-89.22	2013-2014	[32]
35.12	-89.22	2013-2014	
27.15	-81.20	2015-2017	
27.15	-81.20	2015-2017	
27.15	-81.20	2015-2017	
27.15	-81.20	2015-2017	
27.15	-81.20	2015-2017	[79]
27.15	-81.20	2015-2017	
27.15	-81.20	2015-2017	
27.15	-81.20	2015-2017	
27.15	-81.20	2015-2017	
27.15	-81.20	2015-2017	
37.30	-76.61	2010-2014	
36.80	-76.45	2010-2014	
36.63	-76.36	2010-2014	
37.09	-76.35	2010-2014	[80]
36.80	-76.30	2010-2014	
36.91	-76.02	2010-2014	
37.14	-75.97	2010-2014	
36.67	-75.91	2010-2014	
36.66	-76.33	2008-2014	
36.66	-76.33	2008-2014	
36.66	-76.33	2008-2014	
36.66	-76.33	2008-2014	
36.66	-76.33	2008-2014	
36.66	-76.33	2008-2014	
36.80	-76.02	2008-2014	[81]
36.81	-75.96	2008-2014	
36.67	-75.91	2008-2014	
36.67	-75.91	2008-2014	
36.67	-75.91	2008-2014	
36.62	-75.89	2008-2014	
36.62	-75.89	2008-2014	
36.62	-75.89	2008-2014	
35.61	-97.61	2015	
35.61	-97.61	2015	[36]
35.61	-97.61	2015	
35.48	-97.46	2015	

35.48	-97.46	2015	
35.48	-97.46	2015	
38.71	-77.24	2010-2011	[37]
38.71	-77.24	2010-2011	
33.48	-88.84	2005-2007	
30.41	-88.41	2005-2007	
29.98	-84.65	2005-2007	[82]
29.98	-84.65	2005-2007	
30.06	-84.48	2005-2007	
17.10	-88.94	2014-2015	
17.10	-88.94	2014-2015	[83]
17.10	-88.94	2014-2015	
35.11	-89.21	2013	
35.11	-89.21	2013	[41]
35.41	-86.81	2013	
35.41	-86.81	2013	
33.50	-88.96	2012-2014	
33.50	-88.96	2012-2014	[84]
30.43	-88.43	2012-2014	
30.43	-88.43	2012-2014	
31.87	-81.60	1996-1997	[85]
31.87	-81.60	1996-1997	
4.40	-75.14	2014-2016	
4.40	-75.14	2014-2016	
3.93	-74.98	2014-2016	
3.93	-74.98	2014-2016	[86]
5.57	-74.89	2014-2016	
4.46	-75.24	2014-2016	
3.93	-75.02	2014-2016	
5.58	-74.89	2014-2016	
30.65	-96.30	2012-2013	[87]
30.65	-96.30	2012-2013	
20.40	-89.54	2012-2013	[88]
31.15	-97.82	2008-2011	
31.15	-97.82	2008-2011	
31.15	-97.82	2008-2011	
31.15	-97.82	2008-2011	
31.15	-97.82	2008-2011	
28.12	-97.44	2008-2011	
28.12	-97.44	2008-2011	[44]
28.12	-97.44	2008-2011	
28.12	-97.44	2008-2011	
28.12	-97.44	2008-2011	
28.12	-97.44	2008-2011	
28.12	-97.44	2008-2011	
28.12	-97.44	2008-2011	
28.12	-97.44	2008-2011	
28.33	-98.83	1996-1997	[53]
27.52	-97.87	1996-1997	

28.36	-97.21	1985, 1987, 1991		[89]
36.78	-88.07	1982		[90]
36.92	-87.98	1982		
28.36	-97.21	1985-1987		[91]
34.02	-91.35	2000		[92]
35.12	-89.22	2012		[59]
35.12	-89.22	2012		
35.12	-89.22	2012		
35.12	-89.22	2012		
35.12	-89.22	2012		
35.12	-89.22	2012		
35.12	-89.22	2012		
35.12	-89.22	2012		
35.12	-89.22	2012		
35.12	-89.22	2012		
35.12	-89.22	2012		
35.12	-89.22	2012		
35.12	-89.22	2012		
35.12	-89.22	2012		
35.12	-89.22	2012		
35.12	-89.22	2012		
35.12	-89.22	2012		
35.12	-89.22	2012		
35.12	-89.22	2012		
35.12	-89.22	2012		
35.12	-89.22	2012		
35.12	-89.22	2012		
35.12	-89.22	2012		
42.57	-79.04	1979		[93]
35.77	-78.64	1995-2010	VectorMap	[66–68]
37.15	-76.59	1995-2010	VectorMap	
32.50	-93.65	1995-2010	VectorMap	
32.95	-79.95	1995-2010	VectorMap	
30.43	-86.69	1995-2010	VectorMap	
30.63	-87.04	1995-2010	VectorMap	
31.33	-89.29	1995-2010	VectorMap	
34.61	-98.39	1995-2010	VectorMap	
35.50	-76.45	1995-2010	VectorMap	
38.57	-77.33	1995-2010	VectorMap	
27.95	-99.73	1995-2010	VectorMap	
34.01	-77.98	1995-2010	VectorMap	
37.08	-76.37	1995-2010	VectorMap	
38.70	-77.15	1995-2010	VectorMap	
30.14	-85.74	1995-2010	VectorMap	
27.75	-98.08	1995-2010	VectorMap	

33.71	-84.44	1995-2010	VectorMap	
40.72	-89.60	1995-2010	VectorMap	
27.48	-81.56	1995-2010	VectorMap	
29.91	-91.66	1995-2010	VectorMap	
29.29	-82.11	1995-2010	VectorMap	
30.40	-88.89	1995-2010	VectorMap	
31.19	-89.17	1995-2010	VectorMap	
32.05	-96.47	1995-2010	VectorMap	
33.09	-79.46	1995-2010	VectorMap	

* *A. maculatum*: GBIF.org (accessed 17th September 2018) GBIF Occurrence Download https://doi.org/10.15468/dl.vsfgjy

Table S3. Each geolocation recorded for ticks in the species *Amblyomma cajennense* in the literature and publicly available online databases.

Latitude	Longitude	Year	Data Source	Reference
26.31	-109.03	1955		Essig Museum of Entomology
28.03	-97.51	1982		Essig Museum of Entomology
18.09	-96.14	1953		Essig Museum of Entomology
28.03	-97.51	1982		Essig Museum of Entomology
18.13	-96.14	1953		Essig Museum of Entomology
28.03	-97.51	1982		Essig Museum of Entomology
28.03	-97.51	1982		Essig Museum of Entomology
-20.51	-54.62	2018		iNaturalist Research-grade Observation
28.65	-97.38	2017		iNaturalist Research-grade Observation
26.09	-98.14	2017		iNaturalist Research-grade Observation
20.49	-105.44	2016		iNaturalist Research-grade Observation
23.30	-106.44	2016		iNaturalist Research-grade Observation
23.30	-106.44	2014		iNaturalist Research-grade Observation
23.30	-106.44	2013		iNaturalist Research-grade Observation
23.30	-106.44	2013		iNaturalist Research-grade Observation
16.91	-92.09	1950	GBIF*	Illinois Natural History Survey
16.91	-92.09	1950		Illinois Natural History Survey
21.98	-99.02	Not specified		Illinois Natural History Survey
20.67	-88.57	1951		Illinois Natural History Survey
20.67	-88.57	1951		Illinois Natural History Survey
17.07	-92.25	1950		Illinois Natural History Survey
14.94	-89.98	1959		Illinois Natural History Survey
18.66	-95.40	Not specified		Instituto de Diagnóstico y Referencia Epidemiológicos, Mexico
21.98	-99.01	Not specified		Instituto de Diagnóstico y Referencia Epidemiológicos, Mexico
18.66	-95.40	Not specified		Instituto de Diagnóstico y Referencia Epidemiológicos, Mexico
18.66	-95.40	Not specified		Instituto de Diagnóstico y Referencia Epidemiológicos, Mexico
21.59	-105.17	1992		Instituto de Diagnóstico y Referencia Epidemiológicos, Mexico

21.10	-98.46	2009	Instituto de Diagnóstico y Referencia Epidemiológicos, Mexico
20.39	-98.21	2011	Instituto de Diagnóstico y Referencia Epidemiológicos, Mexico
18.66	-95.40	Not specified	Instituto de Diagnóstico y Referencia Epidemiológicos, Mexico
21.37	-98.66	Not specified	Instituto de Diagnóstico y Referencia Epidemiológicos, Mexico
21.10	-98.46	2009	Instituto de Diagnóstico y Referencia Epidemiológicos, Mexico
19.45	-104.36	2007	Instituto de Diagnóstico y Referencia Epidemiológicos, Mexico
21.10	-98.46	2009	Instituto de Diagnóstico y Referencia Epidemiológicos, Mexico
32.66	-115.47	2009	Instituto de Diagnóstico y Referencia Epidemiológicos, Mexico
17.36	-99.47	2012	Instituto de Diagnóstico y Referencia Epidemiológicos, Mexico
18.45	-95.21	Not specified	Instituto de Diagnóstico y Referencia Epidemiológicos, Mexico
19.45	-104.36	2010	Instituto de Diagnóstico y Referencia Epidemiológicos, Mexico
19.15	-96.96	Not specified	Instituto de Diagnóstico y Referencia Epidemiológicos, Mexico
21.26	-98.79	Not specified	Instituto de Diagnóstico y Referencia Epidemiológicos, Mexico
16.82	-95.14	2011	Instituto de Diagnóstico y Referencia Epidemiológicos, Mexico
19.15	-96.96	Not specified	Instituto de Diagnóstico y Referencia Epidemiológicos, Mexico
21.10	-98.46	2009	Instituto de Diagnóstico y Referencia Epidemiológicos, Mexico
21.10	-98.46	2009	Instituto de Diagnóstico y Referencia Epidemiológicos, Mexico
20.59	-100.39	2012	Instituto de Diagnóstico y Referencia Epidemiológicos, Mexico
16.82	-95.14	2011	Instituto de Diagnóstico y Referencia Epidemiológicos, Mexico
21.10	-98.46	2009	Instituto de Diagnóstico y Referencia Epidemiológicos, Mexico
20.39	-98.21	2011	Instituto de Diagnóstico y Referencia Epidemiológicos, Mexico
16.03	-96.62	2008	Instituto de Diagnóstico y Referencia Epidemiológicos, Mexico
21.10	-98.46	2009	Instituto de Diagnóstico y Referencia Epidemiológicos, Mexico
21.37	-98.66	Not specified	Instituto de Diagnóstico y Referencia Epidemiológicos, Mexico
16.82	-95.14	2011	Instituto de Diagnóstico y Referencia Epidemiológicos, Mexico
21.59	-105.17	1992	Instituto de Diagnóstico y Referencia Epidemiológicos, Mexico
16.82	-95.14	2011	Instituto de Diagnóstico y Referencia Epidemiológicos, Mexico
21.37	-98.66	Not specified	Instituto de Diagnóstico y Referencia Epidemiológicos, Mexico
16.82	-95.14	2011	Instituto de Diagnóstico y Referencia Epidemiológicos, Mexico

18.66	-95.40	Not specified		Instituto de Diagnóstico y Referencia Epidemiológicos, Mexico
16.82	-95.14	2011		Instituto de Diagnóstico y Referencia Epidemiológicos, Mexico
-3.14	-60.02	1977		Instituto Nacional de Pesquisas da Amazônia
29.42	-98.49	Not specified		Ohio State University Acarology Laboratory
-11.43	-61.45	2015-2016		
-11.67	-61.19	2015-2016		[94]
-12.73	-60.14	2015-2016		
27.57	-99.43	2005-2008		[95]
-22.69	-47.25	2009-2010		
-22.72	-47.32	2009-2010		
-22.70	-47.30	2009-2010		[96]
-22.69	-47.29	2009-2010		
-22.75	-47.27	2009-2010		
-22.69	-47.29	2009-2010		
-8.38	-35.45	2007-2008		
-8.20	-35.56	2007-2008		[97]
-8.33	-35.71	2007-2008		
-20.44	-54.65	2012		[98]
-19.13	-56.77	1996-1999		[99]
14.04	-90.08	2007		[100]
-18.93	-48.26	2010		[101]
-21.99	-47.42	1997-1999		[102]
14.58	-88.58	Not specified		[103]
-22.68	-42.78	2006		
-22.89	-42.99	2006		[104]
-22.12	-43.17	2006		
-22.20	-44.97	2006	Literature	
-22.12	-43.21	2007-2008		
-22.78	-41.96	2007-2008		
-22.20	-44.97	2007-2008		[105]
29.56	-98.75	2007-2008		
29.81	-95.60	2007-2008		
30.28	-97.90	2007-2008		
-24.90	-64.95	1986-1988		[106]
-5.99	-48.63	2012		
-6.11	-48.59	2012		
-12.39	-49.12	2012		
-7.33	-48.30	2012		
-6.59	-48.64	2012		
-6.86	-48.52	2012		
-9.86	-48.72	2012		
-8.48	-48.48	2012		[107]
-11.04	-48.75	2012		
-11.90	-49.17	2012		
-11.21	-48.93	2012		
-12.23	-49.17	2012		
-8.98	-48.50	2012		
-11.83	-49.13	2012		
-9.42	-48.57	2012		

-7.80	-48.46	2012	
-10.57	-48.92	2012	
-10.91	-48.91	2012	
-5.97	-47.88	2012	
-12.79	-49.09	2012	
-6.25	-47.46	2012	
-6.34	-47.55	2012	
-6.41	-48.54	2012	
-4.52	-46.78	2012	
-6.34	-47.41	2012	
-2.26	-45.27	2012	
-3.88	-45.55	2012	
-4.08	-45.95	2012	
-12.88	-49.11	2012	
-11.87	-61.00	2012	
-11.16	-61.90	2012	
-12.72	-60.26	2012	
-12.49	-60.47	2012	
-13.94	-59.76	2012	
-13.32	-59.88	2012	
-13.00	-59.96	2012	
-14.78	-59.35	2012	
-14.17	-59.70	2012	
-14.13	-59.71	2012	
-14.93	-60.02	2012	
-13.76	-55.87	2013	
-11.93	-55.70	2013	
-13.76	-55.87	2013	
-1.83	-45.38	2012	
-1.67	-47.77	2011	
-6.10	-48.58	2012	
-12.05	-63.57	2011	
-12.73	-60.13	2002	
-10.60	-49.18	2011	
-2.45	-44.78	2012	
-3.73	-43.35	2012	
-2.55	-59.78	2012	
-3.68	-45.40	2012	
-13.08	-59.88	2012	
-10.63	-51.57	2011	
-15.15	-59.47	2012	
-11.87	-55.60	2013	
-18.26	-52.94	1999-2008	[108]
-24.68	-64.60	Not specified	[109]
-25.92	-61.72	Not specified	
-19.00	-56.63	2006-2007	
-18.99	-56.60	2006-2007	
-18.98	-56.64	2006-2007	[110]
-18.98	-56.64	2006-2007	
-18.99	-56.63	2006-2007	

-19.02	-56.66	2006-2007	
-19.02	-56.64	2006-2007	
-18.97	-56.60	2006-2007	
-18.98	-56.64	2006-2007	
-18.98	-56.60	2006-2007	
-18.98	-56.63	2006-2007	
-18.99	-56.62	2006-2007	
-18.98	-56.65	2006-2007	
-18.99	-56.63	2006-2007	
-18.99	-56.66	2006-2007	
-18.99	-56.66	2006-2007	
-18.98	-56.64	2006-2007	
-19.00	-56.63	2006-2007	
-18.99	-56.63	2006-2007	
8.52	-75.84	2009	[111]
-20.07	-50.15	2012	[112]
-21.20	-61.65	2012	
-14.44	-39.17	2009	[113]
-22.53	-52.18	2004-2006	[113]
-19.62	-44.03	1995-1997	[114]
-19.62	-42.03	1995-1997	[115]
-21.70	-43.44	2008	[116]
-21.72	-43.41	2008	
-22.60	-43.71	2012	[117]
-22.60	-43.71	2012	
-19.83	-43.83	1997	[118]
27.65	-98.20	2005-2006	[119]
-18.60	-45.35	2005-2010	[120]
-16.35	-46.90	2005-2010	
-20.61	-42.14	2005-2010	
-19.03	-43.42	2005-2010	
-19.61	-43.22	2005-2010	
-21.20	-51.84	2008-2009	[121]
-21.12	-51.76	2008-2009	
-21.13	-51.77	2008-2009	
-21.15	-51.77	2008-2009	
-21.15	-51.74	2008-2009	
-21.18	-51.79	2008-2009	
-21.23	-51.81	2008-2009	
-21.27	-51.84	2008-2009	
-21.27	-51.84	2008-2009	
-21.29	-51.83	2008-2009	
-22.41	-42.96	2009-2010	[122]
-13.72	-50.79	2004-2005	[123]
-13.72	-50.79	2004-2005	
-13.73	-50.79	2004-2005	
-13.74	-50.79	2004-2005	
-13.74	-50.79	2004-2005	
-13.72	-50.78	2004-2005	
-13.72	-50.78	2004-2005	

-13.75	-50.82	2004-2005		
-15.06	-50.40	2004-2005		
-15.07	-50.40	2004-2005		
-15.06	-50.43	2004-2005		
-15.01	-50.41	2004-2005		
-15.00	-50.40	2004-2005		
-21.12	-51.77	2002-2003		
-21.12	-51.77	2002-2003		
-21.12	-51.77	2002-2003		
-21.12	-51.77	2002-2003		
-21.12	-51.79	2002-2003		[124]
-21.12	-51.79	2002-2003		
-21.11	-51.80	2002-2003		
-21.10	-51.72	2002-2003		
-21.10	-51.71	2002-2003		
-21.08	-51.71	2002-2003		
-21.74	-52.27	Not specified		[125]
-23.32	-51.15	2007		[126]
-23.35	-51.14	2006-2007		[127]
-19.17	-48.38	2006-2008		[128]
-25.92	-61.72	1995-2010		
-18.98	-56.65	1995-2010		
9.72	-82.86	1995-2010		
9.90	-83.68	1995-2010		
5.02	-74.47	1995-2010		
-23.02	-45.55	1995-2010		
-24.68	-64.60	1995-2010		
-10.63	-63.52	1995-2010		
8.62	-80.13	1995-2010		
-21.99	-47.42	1995-2010		
-19.73	-43.26	1995-2010		
27.75	-98.08	1995-2010		
-19.48	-57.01	1995-2010	VectorMap	[66–68]
-23.32	-51.17	1995-2010		
-13.73	-50.79	1995-2010		
-15.01	-50.41	1995-2010		
27.95	-99.73	1995-2010		
28.04	-97.51	1995-2010		
28.04	-97.51	1995-2010		
28.04	-97.51	1995-2010		
16.91	-92.09	1995-2010		
16.91	-92.09	1995-2010		
18.10	-96.12	1995-2010		
24.86	-99.57	1995-2010		
24.86	-99.57	1995-2010		
29.42	-98.49	1995-2010		

* *A. cajennense*: GBIF.org (accessed 3rd January 2019) GBIF Occurrence Download https://doi.org/10.15468/dl.i4dp2i

Table S4. Each geolocation recorded for ticks in the species *Amblyomma mixtum* in the literature and publicly available online databases.

Latitude	Longitude	Year	Data Source	Reference
-98.75	25.80	1995-2010	GBIF*	
-99.73	27.95	Not specified	Literature	[129]
-97.35	26.23	2015		[71]
-92.99	17.97	2014-2015		[130]
-78.74	9.20	2010-2013		[131]
-79.60	9.01	2010-2013		
-79.70	9.12	2010-2013		
-79.96	9.20	2010-2013		
-87.46	15.77	2014		[132]
-70.75	7.07	2014-2016		[86]
-72.20	5.64	2014-2016		
-72.39	5.33	2014-2016		
-73.88	3.37	2014-2016		
-75.52	5.17	2014-2016		
-88.16	21.15	2009-2014		[88]
-88.18	21.02	2009-2014		
-88.20	20.69	2009-2014		
-88.20	20.80	2009-2014		
-88.27	21.30	2009-2014		
-88.60	20.97	2009-2014		
-88.75	20.90	2009-2014		
-88.92	20.12	2009-2014		
-89.05	20.07	2009-2014		
-89.53	20.92	2009-2014		
-89.54	20.40	2009-2014		
-89.63	20.97	2009-2014		
-89.71	20.49	2009-2014		
-105.08	19.55	2013		[133]
-82.83	9.73	2008-2012		[134]
-83.68	9.90	2008-2012		

* *A. mixtum*: GBIF.org (accessed 3rd January 2019). GBIF Occurrence Download https://doi.org/10.15468/dl.izikpz.

References

1. Al-Warid, H.S.; Beringer, J.; Hiller, T.L.; Belant, J.L.; Gompper, M.E. Community composition of Ixodid ticks parasitizing American black bears in Missouri, USA. *Ursus* **2017**, *27*, 61–66.
2. Allan, B.F. Influence of prescribed burns on the abundance of *Amblyomma americanum* (Acari: Ixodidae) in the Missouri Ozarks. *J. Med. Entomol.* **2009**, *46*, 1030–1036.
3. Bacon, R.M.; Gilmore, R.D.; Quintana, M.; Piesman, J.; Johnson, B.J.B. DNA evidence of *Borrelia lonestari* in *Amblyomma americanum* (Acari: Ixodidae) in southeast Missouri. *J. Med. Entomol.* **2003**, *40*, 590–592.
4. Bissinger, B.W.; Apperson, C.S.; Watson, D.W.; Arellano, C.; Sonenshine, D.E.; Roe, R.M. Novel field assays and the comparative repellency of BioUD®, DEET and permethrin against *Amblyomma americanum*. *Med. Vet. Entomol.* **2011**, *25*, 217–226.
5. Bouzek, D.C.; Foré, S.A.; Bevell, J.G.; Kim, H.-J. A conceptual model of the *Amblyomma americanum* life cycle in northeast Missouri. *J. Vector Ecol.* **2013**, *38*, 74–81.

6. Carroll, J.F.; Klun, J.A.; Kramer, M. Similarity in responses of laboratory-reared and field-collected lone star tick (Acari: Ixodidae) nymphs to repellents. *J. Entomol. Sci.* **2008**, *43*, 426–430.
7. Carroll, J.F.; Pound, J.M.; Miller, J.A.; Kramer, M. Sustained control of Gibson Island, Maryland, populations of *Ixodes scapularis* and *Amblyomma americanum* (Acari: Ixodidae) by community-administered 4-Poster deer self-treatment bait stations. *Vector Borne Zoonotic Dis.* **2009**, *9*, 417–421.
8. Castellaw, A.H.; Showers, J.; Goddard, J.; Chenney, E.F.; Varela-Stokes, A.S. Detection of vector-borne agents in lone star ticks, *Amblyomma americanum* (Acari: Ixodidae), from Mississippi. *J. Med. Entomol.* **2010**, *47*, 473–476.
9. Chitwood, M.C.; Swingen, M.B.; Lashley, M.A.; Flowers, J.R.; Palamar, M.B.; Apperson, C.S.; Olfenbuttel, C.; Moorman, C.E.; DePerno, C.S. Parasitology and serology of free-ranging coyotes (*Canis latrans*) in North Carolina, USA. *J. Wildl. Dis.* **2015**, *51*, 664–669.
10. Cilek, J.E.; Olson, M.A. Seasonal distribution and abundance of ticks (Acari: Ixodidae) in northwestern Florida. *J. Med. Entomol.* **2000**, *37*, 439–444.
11. Cully Jr., J.F. Lone star tick abundance, fire, and bison grazing in tallgrass prairie. *J. Range Manag.* **1999**, *52*, 139.
12. Davidson, W.R.; Siefken, D.A.; Creekmore, L.H. Seasonal and annual abundance of *Amblyomma americanum* (Acari: Ixodidae) in central Georgia. *J. Med. Entomol.* **1994**, *31*, 67–71.
13. Durden, L.A.; Luckhart, S.; Mullen, G.R.; Smith, S. Tick infestations of white-tailed deer in alabama. *J. Wildl. Dis.* **1991**, *27*, 606–614.
14. Florin, D.A.; Brinkerhoff, R.J.; Gaff, H.; Jiang, J.; Robbins, R.G.; Eickmeyer, W.; Butler, J.; Nielsen, D.; Wright, C.; White, A.; et al. Additional U.S. collections of the Gulf Coast tick, *Amblyomma maculatum* (Acari: Ixodidae), from the State of Delaware, the first reported field collections of adult specimens from the State of Maryland, and data regarding this tick from surveillance of migratory songbirds in Maryland. *Syst. Appl. Acarol.* **2014**, *19*, 257–262.
15. Ginsberg, H.S.; Ewing, C.P. Comparison of flagging, walking, trapping, and collecting from hosts as sampling methods for northern deer ticks, *Ixodes dammin*, and lone-star ticks, *Amblyomma americanum* (Acari: Ixodidae). *Exp. Appl. Acarol.* **1989**, *7*, 313–322.
16. Goddard, J.; Sumner, J.W.; Nicholson, W.L.; Paddock, C.D.; Shen, J.; Piesman, J. Survey of ticks collected in Mississippi for *Rickettsia*, *Ehrlichia*, and *Borrelia* species. *J. Vector Ecol.* **2003**, *28*, 184–189.
17. Han, G.S.; Stromdahl, E.Y.; Wong, D.; Weltman, A.C. Exposure to *Borrelia burgdorferi* and other tick-borne pathogens in Gettysburg National Military Park, South-Central Pennsylvania, 2009. *Vector Borne Zoonotic Dis.* **2014**, *14*, 227–233.
18. Heise, S.R.; Elshahed, M.S.; Little, S.E. Bacterial diversity in *Amblyomma americanum* (Acari: Ixodidae) with a focus on members of the genus *Rickettsia*. *J. Med. Entomol.* **2010**, *47*, 258–268.
19. Henning, T.C.; Orr, J.M.; Smith, J.D.; Arias, J.R.; Rasgon, J.L.; Norris, D.E. Discovery of filarial nematode DNA in *Amblyomma americanum* in Northern Virginia. *Ticks Tick Borne Dis.* **2016**, *7*, 315–318.
20. Hudman, D.A.; Sargentini, N.J. Detection of *Borrelia*, *Ehrlichia*, and *Rickettsia* spp. in ticks in northeast Missouri. *Ticks and Tick-borne Dis.* **2016**, *7*, 915–921.
21. Hughes, T.H.; Richardson, A.G.; Hoel, D.F.; Mejeoumov, T.; Farooq, M.; Stoops, C.A. Suppression of *Amblyomma americanum* (Ixodida: Ixodidae) for short-term field operations utilizing cypermethrin and lambda-cyhalothrin. *J. Med. Entomol.* **2014**, *51*, 709–712.
22. Ijdo, J.W.; Wu, C.; Magnarelli, L.A.; Stafford, K.C.; Anderson, J.F.; Fikrig, E. Detection of *Ehrlichia chaffeensis* DNA in *Amblyomma americanum* ticks in Connecticut and Rhode Island. *J. Clin. Microbiol.* **2000**, *38*, 4655–4656.
23. Jackson, L.K.; Gaydon, D.M.; Goddard, J. Seasonal activity and relative abundance of *Amblyomma americanum* in Mississippi. *J. Med. Entomol.* **1996**, *33*, 128–131.
24. Jiang, J.; Yarina, T.; Miller, M.K.; Stromdahl, E.Y.; Richards, A.L. Molecular detection of *Rickettsia amblyommii* in *Amblyomma americanum* parasitizing humans. *Vector Borne Zoonotic Dis.* **2010**, *10*, 329–340.
25. Kensinger, B.J.; Allan, B.F. Efficacy of dry ice-baited traps for sampling *Amblyomma americanum* (Acari: Ixodidae) varies with life stage but not habitat. *J. Med. Entomol.* **2011**, *48*, 708–711.
26. Killmaster, L.F.; Loftis, A.D.; Zemtsova, G.E.; Levin, M.L. Detection of bacterial agents in *Amblyomma americanum* (Acari: Ixodidae) from Georgia, USA, and the use of a multiplex assay to differentiate *Ehrlichia chaffeensis* and *Ehrlichia ewingii*. *J. Med. Entomol.* **2014**, *51*, 868–872.
27. Kinzer, D.R.; Presley, S.M.; Hair, J.A. Comparative efficiency of flagging and carbon dioxide-baited sticky traps for collecting the lone star tick, *Amblyomma americanum* (Acarina: Ixodidae). *J. Med. Entomol.* **1990**, *27*, 750–755.
28. Lepitzki, D.A.; Woolf, A.; Bunn, B.M. Parasites of cottontail rabbits of southern Illinois. *J. Parasitol.* **1992**, *78*, 1080–1083.
29. Long, S.W.; Pound, J.M.; Yu, X. *Ehrlichia* prevalence in *Amblyomma americanum*, central Texas. *Emerg. Infect. Dis.* **2004**, *10*, 1342–1343.
30. Maegli, A.; Loy, J.D.; Cortinas, R. Note on *Ehrlichia chaffeensis*, *Ehrlichia ewingii*, and "*Borrelia lonestari*" infection in lone star ticks (Acari: Ixodidae), Nebraska, USA. *Ticks Tick-borne Dis.* **2016**, *7*, 154–158.
31. Mays, S.E.; Houston, A.E.; Trout Fryxell, R.T. Specifying pathogen associations of *Amblyomma maculatum* (Acari: Ixodidae) in western Tennessee. *J. Med. Entomol.* **2016**, *53*, 435–440.

32. Mays, S.E.; Houston, A.E.; Trout Fryxell, R.T. Comparison of novel and conventional methods of trapping ixodid ticks in the southeastern U.S.A. *Med. Vet. Entomol.* **2016**, *30*, 123–134.
33. Mixson, T.R.; Lydy, S.L.; Dasch, G.A.; Real, L.A. Inferring the population structure and demographic history of the tick, *Amblyomma americanum* Linnaeus. *J. Vector Ecol.* **2006**, *31*, 181–192.
34. Monzón, J.D.; Atkinson, E.G.; Henn, B.M.; Benach, J.L. Population and evolutionary genomics of *Amblyomma americanum*, an expanding arthropod disease vector. *Genome Biol. Evol.* **2016**, *8*, 1351–1360.
35. Noden, B.H.; Dubie, T. Involvement of invasive eastern red cedar (*Juniperus virginiana*) in the expansion of *Amblyomma americanum* in Oklahoma. *J. Vector Ecol.* **2017**, *42*, 178–183.
36. Noden, B.H.; Loss, S.R.; Maichak, C.; Williams, F. Risk of encountering ticks and tick-borne pathogens in a rapidly growing metropolitan area in the U.S. Great Plains. *Ticks Tick-borne Dis.* **2017**, *8*, 119–124.
37. Orr, J.M.; Smith, J.D.; Zawada, S.G.; Arias, J.R. Diel and seasonal activity and trapping of ticks (Acari: Ixodidae) in Northern Virginia, U.S.A. *Syst. Appl. Acarol.* **2013**, *18*, 105–111.
38. Ouellette, J.; Apperson, C.S.; Howard, P.; Evans, T.L.; Levine, J.F. Tick-raccoon associations and the potential for Lyme disease spirochete transmission in the coastal plain of North Carolina. *J. Wildl. Dis.* **1997**, *33*, 28–39.
39. Petry, W.K.; Foré, S.A.; Fielden, L.J.; Kim, H.-J. A quantitative comparison of two sample methods for collecting *Amblyomma americanum* and *Dermacentor variabilis* (Acari: Ixodidae) in Missouri. *Exp. Appl. Acarol.* **2010**, *52*, 427–438.
40. Polito, V.J.; Baum, K.A.; Payton, M.E.; Little, S.E.; Fuhlendorf, S.D.; Reichard, M.V. Tick abundance and levels of infestation on cattle in response to patch burning. *Rangeland Ecology & Management* **2013**, *66*, 545–552.
41. Pompo, K.; Mays, S.; Wesselman, C.; Paulsen, D.J.; Trout Fryxell, R.T. Survey of ticks collected from Tennessee cattle and their pastures for *Anaplasma* and *Ehrlichia* species. *J. Parasitol.* **2016**, *102*, 54–59.
42. Ponnusamy, L.; Gonzalez, A.; Treuren, W.V.; Weiss, S.; Parobek, C.M.; Juliano, J.J.; Knight, R.; Roe, R.M.; Apperson, C.S.; Meshnick, S.R. Diversity of *Rickettsiales* in the microbiome of the lone star tick, *Amblyomma americanum*. *Appl. Environ. Microbiol.* **2014**, *80*, 354–359.
43. Rawlings, J.A.; Teltow, G.J. Prevalence of *Borrelia* (Spirochaetaceae) spirochetes in Texas ticks. *J. Med. Entomol.* **1994**, *31*, 297–301.
44. Sanders, D.M.; Schuster, A.L.; McCardle, P.W.; Strey, O.F.; Blankenship, T.L.; Teel, P.D. Ixodid ticks associated with feral swine in Texas. *J. Vector Ecol.* **2013**, *38*, 361–373.
45. Sardelis, M.R.; Neidhardt, K.; Perich, M.J.; Milstrey, E.G.; Harlan, H.J.; Boobar, L.R. Reduction of the *Amblyomma americanum* (Acari: Ixodidae) population at Fort A.P. Hill, Virginia, by aerial application of diazinon granules: correlation of percentage control with received dose. *J. Med. Entomol.* **1989**, *26*, 494–496.
46. Savage, H.; Burkhalter, K.L.; Godsey, M.S.; Panella, N.A.; Ashley, D.C.; Nicholson, W.L.; Lambert, A.J. Bourbon virus in field-collected ticks, Missouri, USA. *Emerg. Infect. Dis.* **2017**, *23*, 2017–2022.
47. Sayler, K.A.; Wamsley, H.L.; Pate, M.; Barbet, A.F.; Alleman, A.R. Cultivation of *Rickettsia amblyommii* in tick cells, prevalence in Florida lone star ticks (*Amblyomma americanum*). *Parasites & Vectors* **2014**, *7*, 270.
48. Sayler, K.A.; Loftis, A.D.; Beatty, S.K.; Boyce, C.L.; Garrison, E.; Clemons, B.; Cunningham, M.; Alleman, A.R.; Barbet, A.F. Prevalence of tick-borne pathogens in host-seeking *Amblyomma americanum* (Acari: Ixodidae) and *Odocoileus virginianus* (Artiodactyla: Cervidae) in Florida. *J. Med. Entomol.* **2016**, *53*, 949–956.
49. Schulze, T.L.; Jordan, R.A.; Schulze, C.J.; Mixson, T.; Papero, M. Relative encounter frequencies and prevalence of selected *Borrelia*, *Ehrlichia*, and *Anaplasma* infections in *Amblyomma americanum* and *Ixodes scapularis* (Acari: Ixodidae) ticks from central New Jersey. *J. Med. Entomol.* **2005**, *42*, 450–456.
50. Schulze, T.L.; Jordan, R.A.; Healy, S.P.; Roegner, V.E.; Meddis, M.; Jahn, M.B.; Guthrie Sr., D.L. Relative abundance and prevalence of selected *Borrelia* infections in *Ixodes scapularis* and *Amblyomma americanum* (Acari: Ixodidae) from publicly owned lands in Monmouth County, New Jersey. *J. Med. Entomol.* **2006**, *43*, 1269–1275.
51. Schulze, T.L.; Jordan, R.A.; White, J.C.; Roegner, V.E.; Healy, S.P. Geographical distribution and prevalence of selected *Borrelia*, *Ehrlichia*, and *Rickettsia* infections in *Amblyomma americanum* (Acari: Ixodidae) in New Jersey. *J. Am. Mosq. Control Assoc.* **2011**, *27*, 236–244.
52. Semtner, P.J.; Howell, D.E.; Hair, J.A. The ecology and behavior of the lone star tick (Acarina: Ixodidae) I. The relationship between vegetative habitat type and tick abundance and distribution in Cherokee Co., Oklahoma. *J. Med. Entomol.* **1971**, *8*, 329–335.
53. Shender, L.A.; Botzler, R.G.; George, T.L. Analysis of serum and whole blood values in relation to helminth and ectoparasite infections of feral pigs in Texas. *J. Wildl. Dis.* **2002**, *38*, 385–394.
54. Stein, K.J.; Waterman, M.; Waldon, J.L. The effects of vegetation density and habitat disturbance on the spatial distribution of ixodid ticks (Acari: Ixodidae). *Geospat. Health* **2008**, *2*, 241–252.
55. Stromdahl, E.Y.; Vince, M.A.; Billingsley, P.M.; Dobbs, N.A.; Williamson, P.C. *Rickettsia amblyommii* infecting *Amblyomma americanum* larvae. *Vector Borne Zoonotic Dis.* **2008**, *8*, 15–24.
56. Tokarz, R.; Williams, S.H.; Sameroff, S.; Sanchez Leon, M.; Jain, K.; Lipkin, W.I. Virome analysis of *Amblyomma americanum*, *Dermacentor variabilis*, and *Ixodes scapularis* ticks reveals novel highly divergent vertebrate and invertebrate viruses. *J. Virol.* **2014**, *88*, 11480–11492.

57. Tokarz, R.; Sameroff, S.; Tagliafierro, T.; Jain, K.; Williams, S.H.; Cucura, D.M.; Rochlin, I.; Monzon, J.; Carpi, G.; Tufts, D.; et al. Identification of novel viruses in *Amblyomma americanum*, *Dermacentor variabilis*, and *Ixodes scapularis* ticks. *mSphere* **2018**, *3*, e00614-17.
58. Trout Fryxell, R.T.; Moore, J.E.; Collins, M.D.; Kwon, Y.; Jean-Philippe, S.R.; Schaeffer, S.M.; Odoi, A.; Kennedy, M.; Houston, A.E. Habitat and vegetation variables are not enough when predicting tick populations in the southeastern United States. *PLOS One* **2015**, *10*, e0144092.
59. Trout Fryxell, R.T.; Hendricks, B.M.; Pompo, K.; Mays, S.E.; Paulsen, D.J.; Operario, D.J.; Houston, A.E. Investigating the adult Ixodid tick populations and their associated *Anaplasma*, *Ehrlichia*, and *Rickettsia* bacteria at a Rocky Mountain spotted fever hotspot in western Tennessee. *Vector-Borne Zoonotic Dis.* **2017**, *17*, 527–538.
60. Whitlock, J.E.; Fang, Q.Q.; Durden, L.A.; Oliver, J.H. Prevalence of *Ehrlichia chaffeensis* (Rickettsiales: Rickettsiaceae) in *Amblyomma americanum* (Acari: Ixodidae) from the Georgia coast and Barrier Islands. *J. Med. Entomol.* **2000**, *37*, 276–280.
61. Williams-Newkirk, A.J.; Rowe, L.A.; Mixson-Hayden, T.R.; Dasch, G.A. Characterization of the bacterial communities of life stages of free living lone star ticks (*Amblyomma americanum*). *PLOS One* **2014**, *9*, e102130.
62. Willis, D.; Carter, R.; Murdock, C.; Blair, B. Relationship between habitat type, fire frequency, and *Amblyomma americanum* populations in east-central Alabama. *J. Vector Ecol.* **2012**, *37*, 373–381.
63. Zhang, X.; Ren, X.; Norris, D.E.; Rasgon, J.L. Distribution and infection frequency of '*Candidatus* Rickettsia amblyommii' in Maryland populations of the lone star tick (*Amblyomma americanum*) and culture in an *Anopheles gambiae* mosquito cell line. *Ticks Tick-borne Dis.* **2012**, *3*, 38–42.
64. Zieman, E.A.; Jiménez, F.A.; Nielsen, C.K. Concurrent examination of bobcats and ticks reveals high prevalence of *Cytauxzoon felis* in southern Illinois. *J. Parasitol.* **2017**, *103*, 343–348.
65. Zimmerman, R.H.; McWherter, G.R.; Bloemer, S.R. Medium-sized mammal hosts of *Amblyomma americanum* and *Dermacentor variabilis* (Acari: Ixodidae) at Land Between the Lakes, Tennessee, and effects of integrated tick management on host infestations. *J. Med. Entomol.* **1988**, *25*, 461–466.
66. Stromdahl, E.Y.; Evans, S.R.; O'Brien, J.J.; Gutierrez, A.G. Prevalence of infection in ticks submitted to the human tick test kit program of the U.S. Army Center for Health Promotion and Preventive Medicine. *J. Med. Entomol.* **2001**, *38*, 67–74.
67. Stromdahl, E.Y.; Williamson, P.C.; Kollars, T.M.; Evans, S.R.; Barry, R.K.; Vince, M.A.; Dobbs, N.A. Evidence of *Borrelia lonestari* DNA in *Amblyomma americanum* (Acari: Ixodidae) removed from humans. *J. Clin. Microbiol.* **2003**, *41*, 5557–5562.
68. Stromdahl, E.Y.; Jiang, J.; Vince, M.; Richards, A.L. Infrequency of *Rickettsia rickettsii* in *Dermacentor variabilis* removed from humans, with comments on the role of other human-biting ticks associated with spotted fever group Rickettsiae in the United States. *Vector Borne Zoonotic Dis.* **2011**, *11*, 969–977.
69. Allerdice, M.E.J.; Beati, L.; Yaglom, H.; Lash, R.R.; Delgado-de la Mora, J.; Licona-Enriquez, J.D.; Delgado-de la Mora, D.; Paddock, C.D. *Rickettsia parkeri* (Rickettsiales: Rickettsiaceae) detected in ticks of the *Amblyomma maculatum* (Acari: Ixodidae) group collected from multiple locations in southern Arizona. *J. Med. Entomol.* **2017**, *54*, 1743–1749.
70. Castellanos, A.A.; Medeiros, M.C.I.; Hamer, G.L.; Morrow, M.E.; Eubanks, M.D.; Teel, P.D.; Hamer, S.A.; Light, J.E. Decreased small mammal and on-host tick abundance in association with invasive red imported fire ants (*Solenopsis invicta*). *Biol. Lett.* **2016**, *12*.
71. Corn, J.L.; Duhaime, R.A.; Alfred, J.T.; Mertins, J.W.; Leland, B.R.; Sramek, R.L.; Moczygemba, J.D.; Shaw, D.W. Survey for ticks on feral swine within a cattle fever tick-infested landscape in Texas, U.S.A. *Syst. Appl. Acarol.* **2016**, *21*, 1564–1570.
72. Florin, D.A.; Jiang, J.; Robbins, R.G.; Richards, A.L. Infection of the Gulf Coast tick, *Amblyomma maculatum* (Acari: Ixodidae), with *Rickettsia parkeri* : first report from the State of Delaware. *Syst. Appl. Acarol.* **2013**, *18*, 27–29.
73. Forrester, D.J.; Mclaughlin, G.S.; Telford, S.R.; Foster, G.W.; Mccown, J.W. Ectoparasites (Acari, Mallophaga, Anoplura, Diptera) of white-tailed deer, *Odocoileus virginianus* from Southern Florida. *J. Med. Entomol.* **1996**, *33*, 96–101.
74. Goddard, J.; Norment, B.R. Notes on the geographical distribution of the Gulf Coast tick, *Amblyomma maculatum* (Koch) [Acari, Ixodidae]. *Entomological news.* **1983**, *94*, 103–104.
75. Goddard, J.; Paddock, C.D. Observations on distribution and seasonal activity of the Gulf Coast tick in Mississippi. *J. Med. Entomol.* **2005**, *42*, 176–179.
76. Goddard, J.; Varela-Stokes, A.; Schneider, J.C. Observations on questing activity of adult Gulf Coast ticks, *Amblyomma maculatum* Koch (Acari: Ixodidae), in Mississippi, U.S.A. *Syst. Appl. Acarol.* **2011**, *16*, 195–200.
77. Kinsey, A.A.; Durden, L.A.; Oliver, J.H. Tick infestations of birds in coastal Georgia and Alabama. *J. Parasitol.* **2000**, *86*, 251–254.
78. Lee, J.K.; Moraru, G.M.; Stokes, J.V.; Wills, R.W.; Mitchell, E.; Unz, E.; Moore-Henderson, B.; Harper, A.B.; Varela-Stokes, A.S. *Rickettsia parkeri* and "*Candidatus* Rickettsia andeanae" in questing *Amblyomma maculatum* (Acari: Ixodidae) From Mississippi. *J. Med. Entomol.* **2017**, *54*, 476–480.

79. Merrill, M.M.; Boughton, R.K.; Lord, C.C.; Sayler, K.A.; Wight, B.; Anderson, W.M.; Wisely, S.M. Wild pigs as sentinels for hard ticks: A case study from south-central Florida. *Int. J. Parasitol. Parasites Wildl.* **2018**, *7*, 161–170.
80. Nadolny, R.M.; Gaff, H.D. Natural history of *Amblyomma maculatum* in Virginia. *Ticks Tick-borne Dis.* **2018**, *9*, 188–195.
81. Nadolny, R.; Gaff, H.; Carlsson, J.; Gauthier, D. Comparative population genetics of two invading ticks: evidence of the ecological mechanisms underlying tick range expansions. *Infect. Genet. Evol.* **2015**, *35*, 153–162.
82. Paddock, C.D.; Fournier, P.-E.; Sumner, J.W.; Goddard, J.; Elshenawy, Y.; Metcalfe, M.G.; Loftis, A.D.; Varela-Stokes, A. Isolation of *Rickettsia parkeri* and identification of a novel spotted fever group *Rickettsia* sp. from Gulf Coast ticks (*Amblyomma maculatum*) in the United States. *Appl. Environ. Microbiol.* **2010**, *76*, 2689–2696.
83. Polsomboon, S.; Hoel, D.F.; Murphy, J.R.; Linton, Y.-M.; Motoki, M.; Robbins, R.G.; Bautista, K.; Briceño, I.; Achee, N.L.; Grieco, J.P.; et al. Molecular Detection and Identification of Rickettsia Species in Ticks (Acari: Ixodidae) Collected From Belize, Central America. *J. Med. Entomol.* **2017**, *54*, 1718–1726.
84. Portugal III, J.S.; Goddard, J. Collections of immature *Amblyomma maculatum* Koch (Acari: Ixodidae) from Mississippi, U.S.A. *Syst. Appl. Acarol.* **2015**, *20*, 20–24.
85. Pung, O.J.; Durden, L.A.; Patrick, M.J.; Conyers, T.; Mitchell, L.R. Ectoparasites and gastrointestinal helminths of southern flying squirrels in southeast Georgia. *J. Parasitol.* **2000**, *86*, 1051–1055.
86. Rivera-Páez, F.A.; Labruna, M.B.; Martins, T.F.; Perez, J.E.; Castaño-Villa, G.J.; Ossa-López, P.A.; Gil, C.A.; Sampieri, B.R.; Aricapa-Giraldo, H.J.; Camargo-Mathias, M.I. Contributions to the knowledge of hard ticks (Acari: Ixodidae) in Colombia. *Ticks Tick Borne Dis.* **2018**, *9*, 57–66.
87. Rodriguez, J.E.; Hamer, S.A.; Castellanos, A.A.; Light, J.E. Survey of a rodent and tick community in east-central Texas. *Southeastern Naturalist* **2015**, *14*, 415–424.
88. Rodríguez-Vivas, R.I.; Apanaskevich, D.A.; Ojeda-Chi, M.M.; Trinidad-Martínez, I.; Reyes-Novelo, E.; Esteve-Gassent, M.D.; Pérez de León, A.A. Ticks collected from humans, domestic animals, and wildlife in Yucatan, Mexico. *Vet. Parasitol.* **2016**, *215*, 106–113.
89. Sleeba, S.B.; Teel, P.D.; Longnecker, M.T.; Strey, O.F. Host selection by questing female *Amblyomma maculatum* Koch, to cattle with feeding male ticks in southern Texas. *Vet. Parasitol.* **2010**, *172*, 105–108.
90. Snoddy, E.L.; Cooney, J.C. A new distribution record for the Gulf Coast tick, *Amblyomma maculatum* (Acari: Ixodidae). *J. Med. Entomol.* **1984**, *21*, 242–242.
91. Teel, P.D.; Hopkins, S.W.; Donahue, W.A.; Strey, O.F. Population dynamics of immature *Amblyomma maculatum* (Acari: Ixodidae) and other ectoparasites on meadowlarks and northern bobwhite quail resident to the coastal prairie of Texas. *J. Med. Entomol.* **1998**, *35*, 483–488.
92. Trout, R.T.; Steelman, C.D.; Szalanski, A.L.; Loftin, K. Establishment of *Amblyomma maculatum* (gulf coast tick) in Arkansas, U.S.A. *The Florida Entomologist* **2010**, *93*, 120–122.
93. Wiedl, S.C. A new distribution record for *Amblyomma maculatum* (Acari: Ixodidae). *J. Med. Entomol.* **1981**, *18*, 170–170.
94. Aguirre, A.A.R.; Garcia, M.V.; Costa, I.N. da; Csordas, B.G.; Rodrigues, V. da S.; Medeiros, J.F.; Andreotti, R. New records of tick-associated spotted fever group *Rickettsia* in an Amazon-Savannah ecotone, Brazil. *Ticks and Tick-borne Dis.* **2018**, *9*, 1038–1044.
95. Beck, D.L.; Zavala, J.; Montalvo, E.O.; Quintana, F.G. Meteorological indicators for *Amblyomma cajennense* and population dynamics in the Tamaulipan Biotic Province in Texas. *J. Vector Ecol.* **2011**, *36*, 135–146.
96. Brites-Neto, J.; Nieri-Bastos, F.A.; Brasil, J.; Duarte, K.M.R.; Martins, T.F.; Veríssimo, C.J.; Barbieri, A.R.M.; Labruna, M.B. Environmental infestation and rickettsial infection in ticks in an area endemic for Brazilian spotted fever. *Rev. Bras. Parasitol. Vet.* **2013**, *22*, 367–372.
97. Dantas-Torres, F. Ticks on domestic animals in Pernambuco, Northeastern Brazil. *Rev. Bras. Parasitol. Vet.* **2009**, *18*, 22–28.
98. de Almeida, R.F.C.; Garcia, M.V.; Cunha, R.C.; Matias, J.; e Silva, E.A.; de Fatima Cepa Matos, M.; Andreotti, R. Ixodid fauna and zoonotic agents in ticks from dogs: first report of *Rickettsia rickettsii* in *Rhipicephalus sanguineus* in the state of Mato Grosso do Sul, mid-western Brazil. *Exp. Appl. Acarol.* **2013**, *60*, 63–72.
99. De Campos Pereira, M.; Szabó, M.P.J.; Bechara, G.H.; Matushima, E.R.; Duarte, J.M.B.; Rechav, Y.; Fielden, L.; Keirans, J.E. Ticks (Acari: Ixodidae) associated with wild animals in the Pantanal region of Brazil. *J. Med. Entomol.* **2000**, *37*, 979–983.
100. Eremeeva, M.E.; Berganza, E.; Suarez, G.; Gobern, L.; Dueger, E.; Castillo, L.; Reyes, L.; Wikswo, M.E.; Abramowicz, K.F.; Dasch, G.A.; et al. Investigation of an outbreak of rickettsial febrile illness in Guatemala, 2007. *Int. J. Infect. Dis.* **2013**, *17*, e304–e311.
101. Heukelbach, J.; Frank, R.; Ariza, L.; de Sousa Lopes, Í.; de Assis e Silva, A.; Borges, A.C.; Limongi, J.E.; de Alencar, C.H.M.; Klimpel, S. High prevalence of intestinal infections and ectoparasites in dogs, Minas Gerais State (southeast Brazil). *Parasitology Research* **2012**, *111*, 1913–1921.
102. Labruna, M.B.; Kasai, N.; Ferreira, F.; Faccini, J.L.H.; Gennari, S.M. Seasonal dynamics of ticks (Acari: Ixodidae) on horses in the state of São Paulo, Brazil. *Vet. Parasitol.* **2002**, *105*, 65–77.

103. Linthicum, K.J.; Logan, T.M.; Bailey, C.L.; Gordon, S.W.; Peters, C.J.; Monath, T.P.; Osorio, J.; Francy, D.B.; Mclean, R.G.; Leduc, J.W.; et al. Venezuelan equine encephalomyelitis virus infection in and transmission by the tick *Amblyomma cajennense* (Arachnida: Ixodidae). *J. Med. Entomol.* **1991**, *28*, 405–409.
104. Machado-Ferreira, E.; Piesman, J.; Zeidner, N.S.; Soares, C.A.. A prevalent alpha-proteobacterium *Paracoccus* sp. in a population of the Cayenne ticks (*Amblyomma cajennense*) from Rio de Janeiro, Brazil. *Genet. Mol. Biol.* **2012**, *35*, 862–867.
105. Machado-Ferreira, E.; Dietrich, G.; Hojgaard, A.; Levin, M.; Piesman, J.; Zeidner, N.S.; Soares, C.A.G. *Coxiella* symbionts in the Cayenne tick *Amblyomma cajennense*. *Microb. Ecol.* **2011**, *62*, 134–142.
106. Mangold, A.J.; Aguirre, D.H.; Gaido, A.B.; Guglielmone, A.A. Seasonal variation of ticks (ixodidae) in *Bos taurus* × *Bos indicus* cattle under rotational grazing in forested and deforested habitats in northwestern Argentina. *Vet. Parasitol.* **1994**, *54*, 389–395.
107. Martins, T.F.; Barbieri, A.R.M.; Costa, F.B.; Terassini, F.A.; Camargo, L.M.A.; Peterka, C.R.L.; de C. Pacheco, R.; Dias, R.A.; Nunes, P.H.; Marcili, A.; et al. Geographical distribution of *Amblyomma cajennense* (*sensu lato*) ticks (Parasitiformes: Ixodidae) in Brazil, with description of the nymph of *A. cajennense* (*sensu stricto*). *Parasit. Vectors* **2016**, *9*.
108. Martins, T.F.; Furtado, M.M.; Jácomo, A.T.D.A.; Silveira, L.; Sollmann, R.; Tôorres, N.M.; Labruna, M.B. Ticks on free-living wild mammals in Emas National Park, Goiás State, central Brazil. *saaa* **2011**, *16*, 201–207.
109. Mastropaolo, M.; Nava, S.; Guglielmone, A.A.; Mangold, A.J. Biological differences between two allopatric populations of*Amblyomma cajennense* (Acari: Ixodidae) in Argentina. *Exp. Appl. Acarol.* **2011**, *53*, 371–375.
110. Medri, Í.M.; Martins, J.R.; Doyle, R.L.; Mourão, G.; Marinho-Filho, J. Ticks (Acari: Ixodidae) from yellow armadillo, Euphractus sexcinctus (Cingulata: Dasypodidae), in Brazil's Pantanal wetlands. *Neotrop. Entomol.* **2010**, *39*, 823–825.
111. Miranda, J.; Mattar, S. Molecular detection of *Rickettsia bellii* and *Rickettsia* sp. strain Colombianensi in ticks from Cordoba, Colombia. *Ticks Tick-borne Dis.* **2014**, *5*, 208–212.
112. Ogrzewalska, M.; Literak, I.; Martins, T.F.; Labruna, M.B. Rickettsial infections in ticks from wild birds in Paraguay. *Ticks Tick-borne Dis.* **2014**, *5*, 83–89.
113. Ogrzewalska, M.; Pacheco, R.C.; Uezu, A.; Richtzenhain, L.J.; Ferreira, F.; Labruna, M.B. Ticks (Acari: Ixodidae) infesting birds in an Atlantic rain forest region of Brazil. *J. Med. Entomol.* **2009**, *46*, 1225–1229.
114. Oliveira, P.R.; Borges, L.M.F.; Leite, R.C.; Freitas, C.M.V. Seasonal dynamics of the Cayenne tick, *Amblyomma cajennense* on horses in Brazil. *Med. Vet. Entomol.* **2003**, *17*, 412–416.
115. Oliveira, P.R.; Borges, L.M.; Lopes, C.M.; Leite, R.C. Population dynamics of the free-living stages of *Amblyomma cajennense* (Fabricius, 1787) (Acari: ixodidae) on pastures of Pedro Leopoldo, Minas Gerais State, Brazil. *Vet. Parasitol.* **2000**, *92*, 295–301.
116. Pacheco, R.C.; Moraes-Filho, J.; Guedes, E.; Silveira, I.; Richtzenhain, L.J.; Leite, R.C.; Labruna, M.B. Rickettsial infections of dogs, horses and ticks in Juiz de Fora, southeastern Brazil, and isolation of *Rickettsia rickettsii* from *Rhipicephalus sanguineus* ticks. *Med. Vet. Entomol.* **2011**, *25*, 148–155.
117. Pinheiro, M. da C.; Lourenço, E.C.; Patrício, P.M.P.; Sá-Hungaro, I.J.B. de; Famadas, K.M. Free-living ixodid ticks in an urban Atlantic Forest fragment, state of Rio de Janeiro, Brazil. *Rev. Bras. Parasitol. Vet.* **2014**, *23*, 264–268.
118. Rojas, R.; Marini, M.Â.; Coutinho, M.T.Z. Wild birds as hosts of *Amblyomma cajennense* (Fabricius, 1787) (Acari: Ixodidae). *Mem. Inst. Oswaldo Cruz* **1999**, *94*, 315–322.
119. Sanders, D.M.; Parker, J.E.; Walker, W.W.; Buchholz, M.W.; Blount, K.; Kiel, J.L. Field collection and genetic classification of tick-borne Rickettsiae and Rickettsiae-like pathogens from South Texas: *Coxiella burnetii* isolated from field-collected *Amblyomma cajennense*. *Ann. N. Y. Acad. Sci.* **2008**, *1149*, 208–211.
120. Saraiva, D.G.; Fournier, G.F.S.R.; Martins, T.F.; Leal, K.P.G.; Vieira, F.N.; Câmara, E.M.V.C.; Costa, C.G.; Onofrio, V.C.; Barros-Battesti, D.M.; Guglielmone, A.A.; et al. Ticks (Acari: Ixodidae) associated with small terrestrial mammals in the state of Minas Gerais, southeastern Brazil. *Exp. Appl. Acarol.* **2012**, *58*, 159–166.
121. Silveira, I.; Martins, T.F.; Olegário, M.M.; Peterka, C.; Guedes, E.; Ferreira, F.; Labruna, M.B. Rickettsial infection in animals, humans and ticks in Paulicéia, Brazil. *Zoonoses and Public Health* **2015**, *62*, 525–533.
122. Spolidorio, M.G.; Andreoli, G.S.; Martins, T.F.; Brandão, P.E.; Labruna, M.B. Rickettsial infection in ticks collected from road-killed wild animals in Rio de Janeiro, Brazil. *J. Med. Entomol.* **2012**, *49*, 1510–1514.
123. Szabó, M.P.J.; Olegário, M.M.M.; Santos, A.L.Q. Tick fauna from two locations in the Brazilian savannah. *Exp. Appl. Acarol.* **2007**, *43*, 73.
124. Szabó, M.P.J.; Castro, M.B.; Ramos, H.G.C.; Garcia, M.V.; Castagnolli, K.C.; Pinter, A.; Veronez, V.A.; Magalhães, G.M.; Duarte, J.M.B.; Labruna, M.B. Species diversity and seasonality of free-living ticks (Acari: Ixodidae) in the natural habitat of wild Marsh deer (*Blastocerus dichotomus*) in Southeastern Brazil. *Vet. Parasitol.* **2007**, *143*, 147–154.
125. Szabó, M.P.J.; Labruna, M.B.; Pereira, M.C.; Duarte, J.M.B. Ticks (Acari: Ixodidae) on wild marsh-deer (*Blastocerus dichotomus*) from southeast Brazil: infestations before and after habitat loss. *J. Med. Entomol.* **2003**, *40*, 268–274.
126. Toledo, R.S.; Tamekuni, K.; Filho, M.F.S.; Haydu, V.B.; Barbieri, A.R.M.; Hiltel, A.C.; Pacheco, R.C.; Labruna, M.B.; Dumler, J.S.; Vidotto, O. Infection by spotted fever Rickettsiae in people, dogs, horses and ticks in Londrina, Parana State, Brazil. *Zoonoses and Public Health* **2011**, *58*, 416–423.

127. Toledo, R.S.; Tamekuni, K.; Silva Filho, M. de F.; Haydu, V.B.; Pacheco, R.C.; Labruna, M.B.; Dumler, J.S.; Vidotto, O. Study of infection by Rickettsiae of the spotted fever group in humans and ticks in an urban park located in the City of Londrina, State of Paraná, Brazil. *Rev. Soc. Bras. Med. Trop.* **2011**, *44*, 313–317.
128. Veronez, V.A.; Freitas, B.Z.; Olegário, M.M.M.; Carvalho, W.M.; Pascoli, G.V.T.; Thorga, K.; Garcia, M.V.; Szabó, M.P.J. Ticks (Acari: Ixodidae) within various phytophysiognomies of a Cerrado reserve in Uberlândia, Minas Gerais, Brazil. *Exp. Appl. Acarol.* **2009**, *50*, 169.
129. Beck, D.L.; Orozco, J.P. Diurnal questing behavior of *Amblyomma mixtum* (Acari: Ixodidae). *Exp. Appl. Acarol.* **2015**, *66*, 613–621.
130. Coronel-Benedett, K.C.; Ojeda-Robertos, N.F.; González-Garduño, R.; Ibañez, F.M.; Rodríguez-Vivas, R.I. Prevalence, intensity and population dynamics of hard ticks (Acari: Ixodidae) on sheep in the humid tropics of Mexico. *Exp. Appl. Acarol.* **2018**, *74*, 99–105.
131. García S, G.G.; Castro, A.M.; Rodríguez, I.; Bermúdez C, S.E. Ixodid ticks of *Hydrochoerus isthmius* Goldman, 1912 (Rodentia: Caviidae) in Panama. *saaa* **2014**, *19*, 404–408.
132. Novakova, M.; Literak, I.; Chevez, L.; Martins, T.F.; Ogrzewalska, M.; Labruna, M.B. Rickettsial infections in ticks from reptiles, birds and humans in Honduras. *Ticks Tick-borne Dis.* **2015**, *6*, 737–742.
133. Sánchez-Montes, S.; Ríos-Muñoz, C.A.; Espinosa-Martínez, D.V.; Guzmán-Cornejo, C.; Berzunza-Cruz, M.; Becker, I. First report of "*Candidatus* Rickettsia amblyommii" in west coast of Mexico. *Ticks Tick-borne Dis.* **2016**, *7*, 1139–1145.
134. Troyo, A.; Moreira-Soto, R.D.; Calderon-Arguedas, Ó.; Mata-Somarribas, C.; Ortiz-Tello, J.; Barbieri, A.R.M.; Avendaño, A.; Vargas-Castro, L.E.; Labruna, M.B.; Hun, L.; et al. Detection of rickettsiae in fleas and ticks from areas of Costa Rica with history of spotted fever group rickettsioses. *Ticks Tick-borne Dis.* **2016**, *7*, 1128–1134.
